# Comparison of Transcriptomic Signatures between Monkeypox-Infected Monkey and Human Cell Lines

**DOI:** 10.1155/2022/3883822

**Published:** 2022-09-01

**Authors:** Do Thi Minh Xuan, I-Jeng Yeh, Chung-Che Wu, Che-Yu Su, Hsin-Liang Liu, Chung-Chieh Chiao, Su-Chi Ku, Jia-Zhen Jiang, Zhengda Sun, Hoang Dang Khoa Ta, Gangga Anuraga, Chih-Yang Wang, Meng-Chi Yen

**Affiliations:** ^1^Graduate Institute of Cancer Biology and Drug Discovery, College of Medical Science and Technology, Taipei Medical University, Taipei 11031, Taiwan; ^2^Department of Emergency Medicine, Kaohsiung Medical University Hospital, Kaohsiung Medical University, Kaohsiung 80708, Taiwan; ^3^Graduate Institute of Clinical Medicine, College of Medicine, Kaohsiung Medical University, Kaohsiung 80708, Taiwan; ^4^Department of Neurosurgery, Taipei Medical University Hospital, Taipei 11031, Taiwan; ^5^Department of Surgery, School of Medicine, College of Medicine, Taipei Medical University, Taipei 11031, Taiwan; ^6^Ph.D. Program for Cancer Molecular Biology and Drug Discovery, College of Medical Science, Taipei Medical University, Taipei 11031, Taiwan; ^7^School of Medicine, College of Medicine, Taipei Medical University, Taipei 11031, Taiwan; ^8^Emergency Department, Huashan Hospital North, Fudan University, Shanghai 201508, China; ^9^Kaiser Permanente, Northern California Regional Laboratories, The Permanente Medical Group, 1725 Eastshore Hwy, Berkeley, CA 94710, USA; ^10^Department of Statistics, Faculty of Science and Technology, PGRI Adi Buana University, East Java, Surabaya 60234, Indonesia; ^11^TMU Research Center of Cancer Translational Medicine, Taipei Medical University, Taipei 11031, Taiwan

## Abstract

Monkeypox virus (MPV) is a smallpox-like virus belonging to the genus *Orthopoxvirus* of the family Poxviridae. Unlike smallpox with no animal reservoir identified and patients suffering from milder symptoms with less mortality, several animals were confirmed to serve as natural hosts of MPV. The reemergence of a recently reported monkeypox epidemic outbreak in nonendemic countries has raised concerns about a global outburst. Since the underlying mechanism of animal-to-human transmission remains largely unknown, comprehensive analyses to discover principal differences in gene signatures during disease progression have become ever more critical. In this study, two MPV-infected *in vitro* models, including human immortal epithelial cancer (HeLa) cells and rhesus monkey (*Macaca mulatta*) kidney epithelial (MK2) cells, were chosen as the two subjects to identify alterations in gene expression profiles, together with co-regulated genes and pathways that are affected during monkeypox disease progression. Using Gene Ontology (GO), Kyoto Encyclopedia of Genes and Genomes (KEGG), and MetaCore analyses, we discovered that elevated expression of genes associated with interleukins (ILs), G protein-coupled receptors (GPCRs), heat shock proteins (HSPs), Toll-like receptors (TLRs), and metabolic-related pathways play major roles in disease progression of both monkeypox-infected monkey MK2 and human HeLa cell lines. Interestingly, our analytical results also revealed that a cluster of differentiation 40 (CD40), plasmin, and histamine served as major regulators in the monkeypox-infected monkey MK2 cell line model, while interferons (IFNs), macrophages, and neutrophil-related signaling pathways dominated the monkeypox-infected human HeLa cell line model. Among immune pathways of interest, apart from traditional monkeypox-regulated signaling pathways such as nuclear factor- (NF-*κ*B), mitogen-activated protein kinases (MAPKs), and tumor necrosis factors (TNFs), we also identified highly significantly expressed genes in both monkey and human models that played pivotal roles during the progression of monkeypox infection, including *CXCL1*, *TNFAIP3*, *BIRC3*, *IL6*, *CCL2*, *ZC3H12A*, *IL11*, *CSF2*, *LIF*, *PTX3*, *IER3*, *EGR1*, *ADORA2A*, and *DUOX1*, together with several epigenetic regulators, such as histone cluster family gene members, *HIST1H3D*, *HIST1H2BJ*, etc. These findings might contribute to specific underlying mechanisms related to the pathophysiology and provide suggestions regarding modes of transmission, post-infectious sequelae, and vaccine development for monkeypox in the future.

## 1. Introduction

Monkeypox virus (MPV/MPXV) is commonly known as one of the rare systemic infections caused by one specie of the genus *Orthopoxvirus* in the family Poxviridae [1, 2]. Other than the smallpox virus (variola virus (VARV)), the best-known species of this genus, monkeypox virus, together with cowpox virus (CPXV), horsepox virus (HSPV), raccoonpox virus (RCNV), camelpox virus (CMLV), mousepox virus (also referred to as Ectromelia virus (ECTV)), and Alaskapox (AKPV) virus, are species believed to infect healthy humans through different modes of zoonotic transmission [3–5]. After the eradication of smallpox, a result of universal vaccination, almost no natural outbreaks related to *Orthopoxvirus* have been recorded until recently, after a significant number of confirmed monkeypox cases were reported worldwide [6]. The first discovery of this virus in monkeys in the lab (in 1958), followed by the first human infection detected in Africa (in 1970), meant that monkeypox was no longer endemic in animals as previously assumed; at least 400 human cases scattered across the western and central parts of the African continent have been diagnosed with similar clinical characteristics to smallpox but causing milder symptoms and being less lethal [7–9]. As of May 2022, multiple cases of MPV have been documented in at least 11 regions outside of Africa, including the European Zone, Britain, Middle East, Australia, the North American continent (including the United States, Canada, and South America), resulting in unprecedented outbreaks in those developed countries [8–10]. Further, public health investigations revealed the approximately 20-fold increase in incidences that resulted from the discontinuation of smallpox vaccination since 1980, while the probable source of infection could be either an animal reservoir (mainly rodents) or cross-infection [11, 12]. Transmission through person-to-person contact is claimed to primarily occur through close and prolonged contact with fluids from skin lesions, respiratory droplets, fomites of infected people, and even during sexual intercourse among populations of men who have sex with men (MSM) [13, 14].

To date, definite diagnoses of monkeypox are largely based on either a polymerase chain reaction (PCR) of skin lesion specimens or immunohistochemical (IHC) staining of biopsies taken from those infected, as misdiagnoses of other blister-like diseases such as smallpox and chickenpox may occur in cases where only a visual skin examination and/or dermoscopy are solely employed [15, 16]. Although the genomes of several variants of the MPV have been fully sequenced, there are limited reports on the underlying mechanisms of viral transmission and pathogenic pathways of this disease [17, 18]. Moreover, proven treatment and preventive vaccines specifically designed for monkeypox have not yet been formally approved by the agencies, thus clinical care only focuses on symptomatic treatment. Therefore, developing a research design that focuses on the underlying molecular mechanisms during disease progression would greatly contribute to improving the situation as it provides necessary background knowledge for subsequent studies, such as diagnostic test development, epidemiological tracking, designs of vaccines and antiviral drugs, and other related adjuvant treatments.

**Figure 1:**
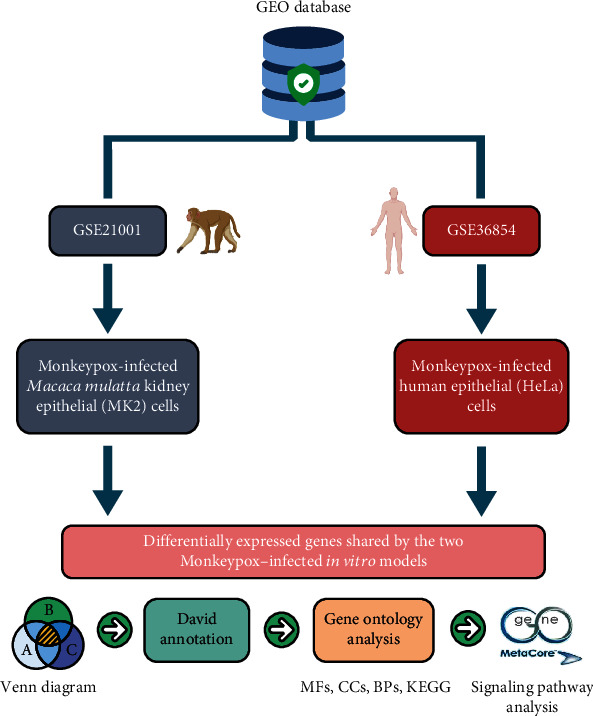
Schematic illustration of the study design. High-throughput data of monkeypox-infected 7-hours of *Macaca mulatta* kidney epithelial (MK2) cells and monkeypox-infected 6-hours of human immortal epithelial cancer (HeLa) cells were both acquired from the GEO database. The top 5% of differentially expressed genes in the two monkeypox-infected models were determined through a Venn diagram analysis. Results of pathway analyses and functional interpretations were analyzed using DAVID, GO, STRING, and MetaCore.

In this study, we attempted to provide a concise and informative overview of principal differences in gene expression profiles between two MPV-infected *in vitro* models, including human immortal epithelial cancer (HeLa) cells and rhesus monkey (*Macaca mulatta*) kidney epithelial (MK2) cells, together with co-regulated genes and pathways that are affected during disease progression. The Gene Expression Omnibus (GEO) database and bioinformatics approaches were employed to perform analyses of available samples related to these two models (Figure 1). We also revealed several critical regulatory downstream networks and investigated the functions of potential genes to determine whether they can serve as clinical biomarkers for MPV. These findings may contribute to an understanding of specific underlying mechanisms related to the pathophysiology and provide suggestions regarding modes of transmission, post-infection sequelae, and vaccine development for monkeypox in the future.

## 2. Materials and Methods

### 2.1. Bioinformatics Application for Data Acquisition and Processing

In this study, we investigated an *in vitro* system using gene expression data of two separate subjects, including human immortal epithelial (HeLa) cells and monkey *Macaca mulatta* kidney epithelial (MK2) cells to characterize the effects of physical monkeypox viral infection. Relevant microarray gene expression datasets from the GEO (http://www.ncbi.nlm.nih.gov/geo)—an international public functional genomics data repository affiliated with the National Center for Biotechnology Information (NCBI; https://www.ncbi.nlm.nih.gov/)—were retrieved for subsequent analyses. In particular, MPV-induced alterations in transcriptomic profiles of 7-hour monkeypox-infected *Macaca mulatta* kidney epithelial (MK2) cells were obtained from the GSE21001 dataset [19], whereas similar information of 6-hour monkeypox-infected human immortal epithelial (HeLa) cells was acquired from the GSE36854 dataset [20]. The following analyses of both studies are based on comparison of each subject with its corresponding mock-infected counterpart as control.

Before being mapped onto Ensembl features using packages attached to the biomaRt tool (vers. 2.26.1), the CLC Genomics Workbench vers. 10.1 (CLC bio, Aarhus, Denmark) was utilized to process and standardize the raw data [21–23]. Signals were subsequently presented as a clustered heatmap based on messenger (m)RNA expression patterns using pheatmap (vers. 1.0.12) in the R environment [24–26], as well as the online platform available at http://www.bioinformatics.com.cn/srplot. For subsequent discoveries of enriched functionally related gene groups, the Database for Annotation, Visualization, and Integrated Discovery (DAVID), which is maintained by the Laboratory of Human Retrovirology and Immunoinformatic (LHRI) [27–29], was chosen as a powerful tool by the various functions it offers, especially in term of Gene Ontology (GO) and Kyoto Encyclopedia of Genes and Genomes (KEGG). As previously described [30–32], the top 5% of upregulated genes derived from monkeypox-infected groups were further processed as input data for the GO analyses [33–35], while a *p* value of <0.05 was set as the significance level. After biological networks and related pathways were constructed, a cut-off value of 0.05 was applied to select significantly enriched pathways or groups of annotated genes.

### 2.2. Pathway-Based Analyses and Network Enrichment Analyses

MetaCore (Enrichment Analysis Workflow and analysis network; GeneGo, St. Joseph, MN, USA) was designed to identify biological processes related to gene microarray data. The top 5% of upregulated genes with substantial differences in transcriptome levels were processed as input to the MetaCore program to compare average levels of gene expressions in the two models. Signal transduction pathways were examined. A statistically significant difference was indicated by a *p* value of <0.05.

### 2.3. Construction of a Protein-Protein-Interacting (PPI) Network using the STRING Analysis

The STRING database (vers. 11.0) (https://string-db.org/) serves as a search engine and a protein information resource that provides a considerable number of proteins and known interaction activities upon which differentially expressed genes (DEGs) were examined [36–38]. The Cytoscape stringApp was also employed in this work to construct a PPI-network, while protein cluster classification was built using the *k*-means clustering algorithm.

## 3. Results

### 3.1. GO Analysis of Monkeypox-Infected Models

**Figure 2:**
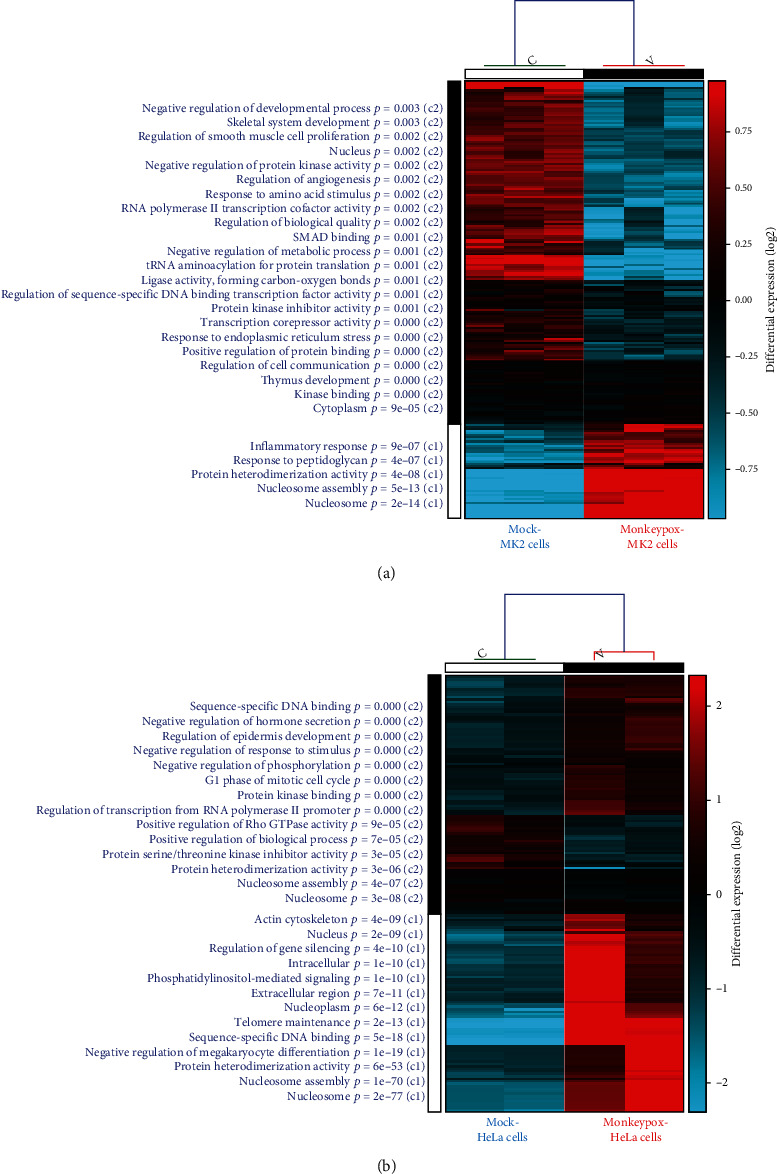
Analytical results of Gene Ontology (GO) enrichment accompanied by heatmap visualization in two separate monkeypox-infected models. (a) Comparison of gene expression patterns between the monkeypox-infected *Macaca mulatta* kidney epithelial (MK2) cells and the mock control. Cluster 1 (c1) contained the most significant highly expressed genes and pathways in the monkeypox-infected group, whereas c2 exerted an opposite trend. The GO analysis suggested that inflammatory and chemokine-related peptidoglycan pathways were significantly correlated with the monkeypox-infected *Macaca mulatta* kidney epithelial (MK2) cell model. (b) Comparison of gene expression patterns between the monkeypox-infected human immortal epithelial cancer (HeLa) cell model and the mock control. Cluster 1 (c1) contained the most significantly highly expressed genes and pathways in the monkeypox-infected group, whereas c2 exerted an opposite trend. The GO analysis suggested that nucleosome assembly and cytoskeleton-related pathways were found to be significantly correlated with the human immortal epithelial cancer (HeLa) cell model.

For the monkey cell line model, transcriptomic data of the 7-hour monkeypox-infected *Macaca mulatta* kidney epithelial (MK2) cell retrieved from the GSE21001 dataset were matched with a mock treatment group for a comparative analysis of DEGs. GO analytical results associated with monkeypox infection are depicted in Figure 2(a). Pathways assigned to cluster 1 (c1) contained the most significant highly expressed genes and pathways in the monkeypox-infected group, whereas cluster 2 (c2) exerted an opposite trend. The GO analysis suggested that c1 was related to inflammatory responses, chemokine activities, immune system processes, responses to peptidoglycans, cellular responses to lipopolysaccharide, nucleosome assembly, chromatin assembly or disassembly, responses to ionizing radiation, and responses to temperature stimuli. Pathways within c2 were associated with kinase binding, thymus development, responses to endoplasmic reticular stress, transfer (t)RNA aminoacylation for protein translation, and responses to amino acid stimulation.

For the 6-hour monkeypox-infected human cell line model, transcriptomic data of monkeypox-infected human HeLa cells were retrieved from the GSE36854 dataset. Pathways assigned to cluster 1 (c1) were those most highly correlated with monkeypox infection, whereas cluster 2 (c2) exerted an opposite trend. The GO analysis suggested that pathways within c1 were related to growth factor activities, leukocyte activation, responses to organic cyclic compounds, inflammatory responses, regulation of the extracellular signal-regulated kinase 1 (ERK1) and the extracellular signal-regulated kinase 2 (ERK2) cascade, chemokine activities, and regulation of nitric oxide biosynthetic processes. Pathways in c2 were related to nucleosome assembly, protein heterodimerization activities, telomere maintenance, extracellular regions, the actin cytoskeleton, responses to protozoans, and coagulation.

### 3.2. Pathway Analysis of the Two Monkeypox-Infected Models

**Figure 3:**
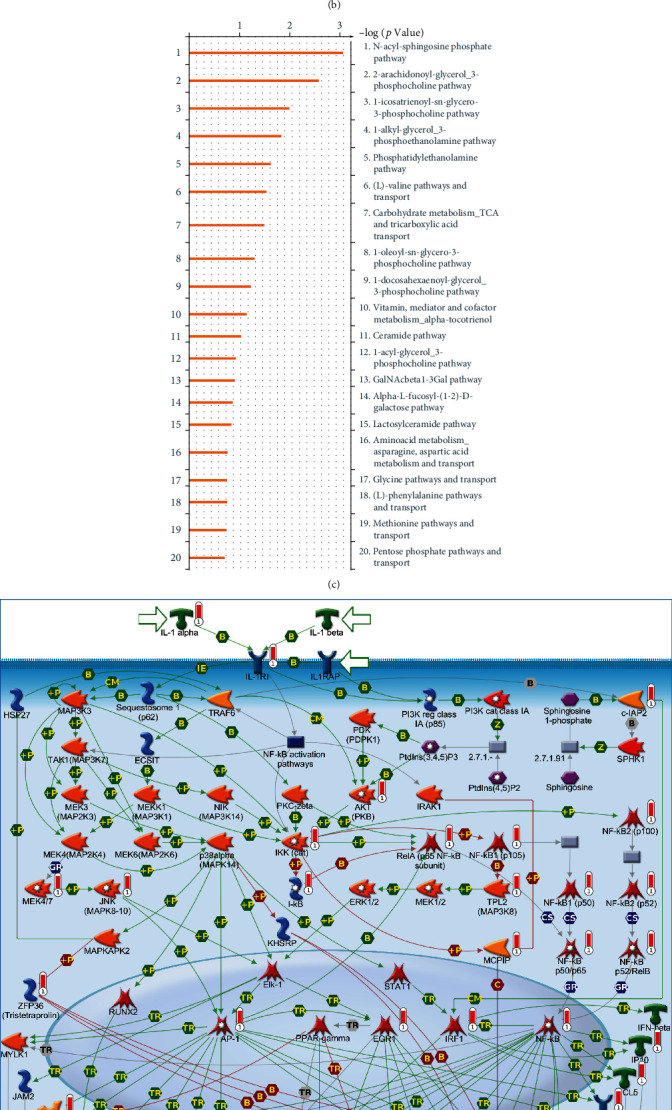
Overexpressed pathways shared by the two *in vitro* monkeypox-infected models. (a) The top 5% of overexpressed genes shared by the GSE21001 and GSE36854 datasets, filtered by a Venn diagram, represent the monkeypox-infected *Macaca mulatta* kidney epithelial (MK2) cell model and the human immortal epithelial cancer (HeLa) cell model. (b) The most enriched biological pathways corresponding to genes from the shortlist of upregulated genes shared by the two models, in order of decreasing log (*p* values). (c) The most enriched metabolic pathways as components of the Endogenous Metabolic Networks corresponding to genes from the shortlist of upregulated genes shared by the two models, in order of decreasing log (*p* values). (d) Related pathways and network analyses by MetaCore confirmed the vital role of the “immune response_IL-1 signaling” pathway in both monkeypox-infected models. (e) Endogenous Metabolic Networks analyses by MetaCore confirmed the vital role of the “N-acyl-sphingosine phosphate pathway” in both monkeypox-infected models.

Venn diagrams were employed to filter upregulated genes in common between the two models mentioned above. The top 5% of messenger (m)RNAs within the monkeypox-infected *Macaca mulatta* kidney epithelial (MK2) cell model and the human immortal epithelial cancer (HeLa) counterpart were highly expressed compared to those within the mock control (Figure 3(a)). In total, 149 overlapping genes that were highly expressed in both monkey and human models were subsequently input to the MetaCore tool for a map analysis. Several standard maps were found to be related to monkeypox infection, including the “immune response_IL-1 signaling pathway,” “NF-*κ*B pathway in multiple myeloma,” “immune response_CD40 signaling in B cells,” “TNF-alpha-induced inflammation,” “signaling in normal and asthmatic airway epithelium,” “immune response_plasmin signaling,” “glomerular injury in lupus nephritis,” “immune response_IL-33 signaling pathway,” “macrophage and dendritic cell phenotype shift in cancer,” “renal tubulointerstitial injury in lupus nephritis,” “signal transduction_nonapoptotic FasR (CD95) signaling,” and “inflammatory mechanisms of pancreatic cancerogenesis” (Figure 3(b), Supplementary Table A1). Meanwhile, Endogenous Metabolic Networks corresponding to genes from the shortlist of upregulated genes shared by the two models revealed that pathways were associated with phospholipid biosynthesis, including the “Ceramide pathway,” “N-acyl-sphingosine phosphate pathway,” “2-arachidonoyl-glycerol 3-phosphocholine pathway,” “phosphatidylethanolamine pathway,” “(L)-valine pathways and transport,” “carbohydrate metabolism_TCA and tricarboxylic acid transport,” and “vitamin, mediator and cofactor metabolism_alpha-tocotrienol,” which are metabolic signalling pathways dominated by these genes within both aforementioned models (Figures 3(c)–3(e)), Supplementary Table A2).

To further understand biological processes in the individual models, we analyzed pathways related to monkeypox infection in each case. For the 7-hour monkeypox-infected *Macaca mulatta* kidney epithelial (MK2) cell model, the top 5% of upregulated genes obtained compared to the mock treatment group were input into the MetaCore platform for a comprehensive map analysis. The “immune response_IL-1 signaling pathway,” “immune response_IL-33 signaling pathway,” “signal transduction_nonapoptotic FasR (CD95) signaling,” “immune response_histamine H1 receptor signaling in immune response,” “IL-1 signaling in melanoma,” “inflammatory mechanisms of pancreatic cancerogenesis,” “immune response_HSP60 and HSP70/TLR signaling pathway,” “G protein-coupled receptors signaling in lung cancer,” “TNF-alpha-induced inflammatory signaling in normal and asthmatic airway epithelium,” and “resistance of pancreatic cancer cells to death receptor signaling” were among the most significant maps detected (Supplementary Figure A1, Table A3). Meanwhile, the endogenous Metabolic Networks corresponding to genes from the shortlist of upregulated genes within the monkeypox-infected *Macaca mulatta* kidney epithelial (MK2) cells revealed that pathways associated with phospholipid biosynthesis and amino acid synthesis included the “phosphatidylethanolamine pathway,” “N-acyl-sphingosine phosphate pathway,” “lyso-phosphatidylserine pathway,” “1-alkyl-glycerol_3-phosphoethanolamine pathway,” “1-oleoyl-glycerol_3-phosphate pathway,” “2-oleoyl-glycerol_3-phosphate pathway,” and “aminoacid metabolism_asparagine, and aspartic acid metabolism and transport,” which are metabolic signaling pathways dominated by these genes within this model (Supplementary Figure A2, Table A4).

For the human immortal epithelial cancer (HeLa) cell model, the top 5% of upregulated genes compared to the mock treatment groups were input into the MetaCore platform for a comprehensive map analysis. The “immune response_IL-1 signaling pathway,” “inflammatory mechanisms of pancreatic cancerogenesis,” “G protein-coupled receptors signaling in lung cancer,” “immune response_plasmin signaling,” “immune response_CD40 signaling in dendritic cells, monocytes, and macrophages,” “interleukins-induced inflammatory response in asthmatic airway fibroblasts,” “TNF-alpha-induced inflammatory signaling in normal and asthmatic airway epithelium,” “NF-*κ*B-, AP-1-, and MAPKs-mediated proinflammatory cytokine production by eosinophils in asthma,” “immune response_CD40 signaling in B cells,” and “immune response_IL-17 signaling pathways” were among the most significant maps detected (Supplementary Figure A3, Table A5). Through the Metabolic Networks (Endogenous) corresponding to genes from the shortlist of upregulated genes shared by the monkeypox-infected human immortal epithelial cancer (HeLa) cells, data revealed that pathways associated with phospholipid biosynthesis included the “phosphatidylcholine pathway,” “1-docosahexaenoyl-glycerol_3-phosphocholine pathway,” “lysophosphatidic acid pathway,” “1-icosatrienoyl-sn-glycero-3-phosphocholine pathway,” “2-arachidonoyl-glycerol_3-phosphocholine pathway,” and “GalNAcbeta1-3Gal pathway,” which are metabolic signaling pathways dominated by these genes within this model (Supplementary Figure A4, Table A6).

In addition to the pathways and network analysis for leading-edge genes generated by MetaCore as mentioned before, we also used the KEGG database to validate these data. The top enriched KEGG pathways were analyzed for common genes shared by the monkeypox-infected *Macaca mulatta* kidney epithelial (MK2) cell model and the human immortal epithelial cancer (HeLa) cell model, and pathways were ranked by *p* values (Supplementary Figure A5).

### 3.3. Analytical Results of Co-regulated Interactions and Cellular Component Annotations in the Monkeypox-Infected Models

**Figure 4:**
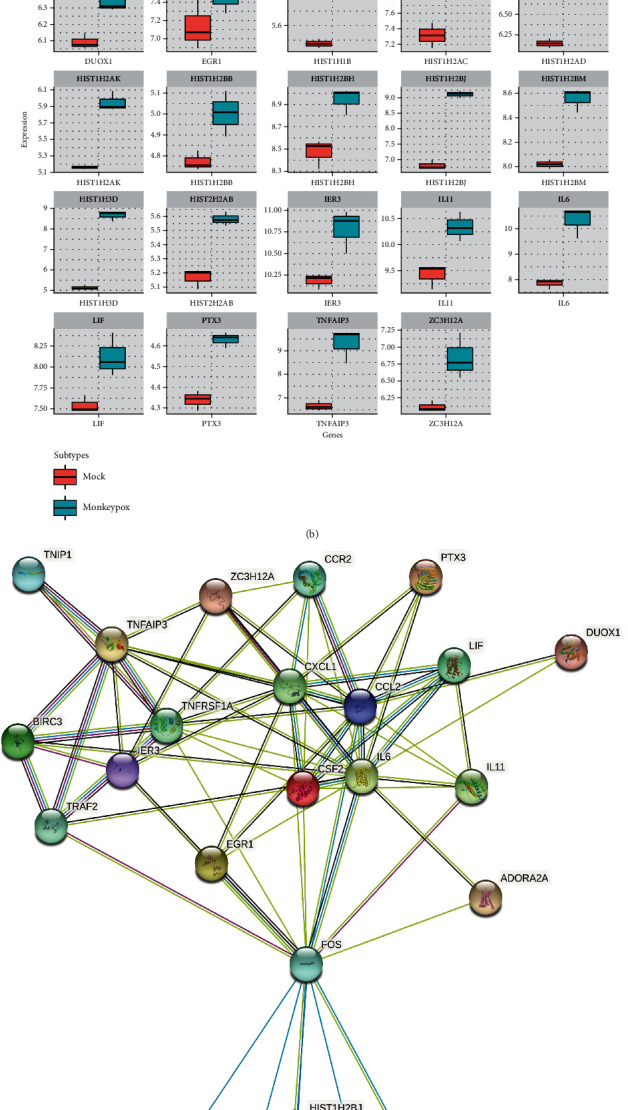
Overview of protein-protein-interacting networks inferred from overexpressed genes in the monkeypox-infected *Macaca mulatta* kidney epithelial (MK2) cell model and the human immortal epithelial cancer (HeLa) cell model. (a) Similar transcriptome patterns obtained from the GSE21001 and GSE36854 datasets were filtered by a Venn diagram. Shortlist of the top 1% of upregulated genes shared by the two monkeypox-infected models. (b) Distribution of 24 coregulated genes inferred from the above shortlist, presented using box plot visualizations. (c) STRING analyses revealed the protein-protein-interacting network of the 24 co-regulated genes, separated by clusters in different colors. Colored nodes represent 24 target genes as input, while gray nodes represent the corresponding proteins.

To further explore the most significance of co-upregulated genes from both models, we used a Venn diagram to filter the 24 genes (among the top 1% of genes) in common, which were highly expressed in both models (Figure 4(a)). Upregulated genes in monkeypox-infected cells included *CXCL1*, *TNFAIP3*, *BIRC3*, *IL-6*, *CCL2*, *ZC3H12A*, *IL-11*, *CSF2*, *LIF*, *PTX3*, *IER3*, *EGR1*, *ADORA2A*, *DUOX1*, and histone clusters family members, such as *HIST1H3D*, *HIST1H2BJ*, *HIST1H2AK*, *HIST1H2AD*, *HIST1H2AC*, *HIST1H1B*, *HIST2H2AB*, *HIST1H2BM*, *HIST1H2BH*, and *HIST1H2BB* (Figure 4(b)). These 24 genes were further imported into the STRING platform to construct PPI networks, which may play pivotal roles in manifestations after monkeypox infection (Figure 4(c)).

**Figure 5:**
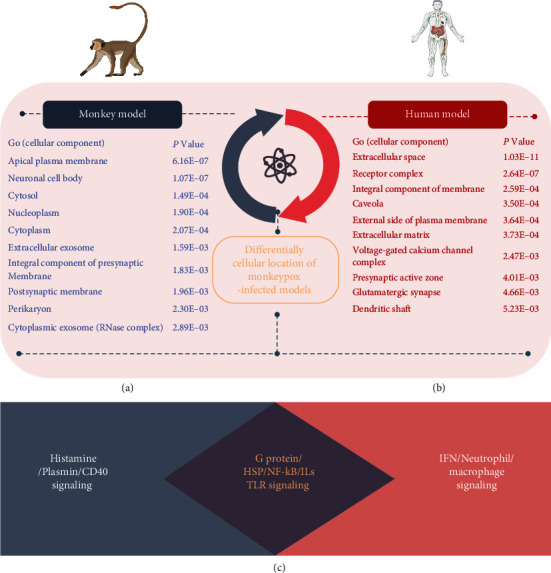
Major characteristics of Gene Ontology (GO) and cellular component annotations are inferred from highly expressed genes shared by the monkeypox-infected *Macaca mulatta* kidney epithelial (MK2) cell model and the human immortal epithelial cancer (HeLa) cell model. (a) Top highly enriched GO cellular components in the MK2 model. (b) Top highly enriched GO cellular components in the HeLa model. (c) Conclusion of enriched maps discovered by bioinformatic analysis of upregulated genes from the MK2 and HeLa models. The data revealed that interleukins (ILs), G protein-coupled receptors (GPCRs), heat shock proteins (HSPs), Toll-like receptors (TLRs), and metabolic-related pathways were the elevated expression of both monkeypox-infected monkey and human cell lines. Besides, cluster of differentiation 40 (CD40), plasmin, and histamine serve as major regulators in the monkeypox-infected monkey cell line model, while interferons (IFNs), macrophages, and neutrophil-related signaling dominate the monkeypox-infected human cell line model.

Upregulated genes shared by these two models were also imported into the GO platform for cellular component annotations under monkeypox infection circumstances. In the case of the monkeypox-infected *Macaca mulatta* kidney epithelial (MK2) cell model, these genes mostly functioned in apical plasma membranes, neuronal cell bodies, the cytosol, nucleoplasm cytoplasm, extracellular exosomes, an integral component of presynaptic membranes, postsynaptic membranes, cytoplasmic exosomes, and the perikaryon (Figure 5(a)). The same analytical flowchart was applied to the monkeypox-infected human immortal epithelial cancer (HeLa) cell model to identify cellular components under monkeypox infection circumstances. Genes with the greatest significance functioned in the extracellular space, receptor complexes, integral component of membranes, caveolae, external side of plasma membranes, extracellular matrices, voltage-gated calcium channel complexes, presynaptic active zones, glutamatergic synapses, and dendritic shafts (Figure 5(b)). From such observations gained from the two *in vitro* monkeypox-infected models, we concluded that certain cellular mechanisms of monkeypox infection are almost identical but slightly different when comparing the two species (Figure 5(c)).

## 4. Discussion

In most cases, viral infections only occur when a virus that causes disease successfully invades a host, beginning by penetrating the antiviral defenses, inserting its genetic materials, taking control of host cells' machinery to rapidly create a huge number of viral replications, and ultimately becoming transmissible between individuals. In the context of the world having gone through many epidemic zoonotic outbreaks that originated from animals [39–41], including the human immunodeficiency virus (HIV; which jumped from chimpanzees), influenza (from birds and pigs), bovine spongiform encephalopathy (BSE; from cows), Ebola (from bats), and at least three zoonotic coronavirus diseases in chronological order, including the severe acute respiratory syndrome coronavirus (SARS-CoV), the Middle Eastern respiratory syndrome coronavirus (MERS-CoV), and the severe acute respiratory syndrome coronavirus-2 (SARS-CoV-2). Therefore, gaining a comprehensive understanding of the mechanisms of infection in both human and non-human hosts is urgently needed [42–45].

In attempts to prevent further outbreaks and deaths from these diseases, worldwide efforts to discover rapid early-detection methods, effective antiviral drugs, and preventive vaccines have been increasing over the years [46, 47]. Other than conventional approaches, novel and advantageous technologies, such as high-throughput sequencing, searchable database repositories of gene expression data, and assistance from web-based or computer-based bioinformatics tools, have been employed to elucidate underlying molecular mechanisms during viral infection and explore how different host cells respond via performing analyses of alterations in gene expressions [48–54].

In the case of monkeypox infection, according to previous studies, a considerable number of immunomodulation-related cytokines, including interleukin-1 (IL-1), tumor necrosis factors (TNFs), and interferons (IFNs), were reported to be involved in disease progression. The ILs superfamily (including IL-1, IL-18, and IL-33) and TNF signaling-mediated immune responses are important for antiviral activity [55, 56]; however, the roles of these signaling pathways in monkeypox-infected somatic cells remain unclear. Interestingly, in case of other zoonotic viral diseases mentioned before, numerous cellular and molecular immunomodulatory pathways were also proven to play crucial roles. In particular, the G protein-coupled receptors (GPCRs) not only helps stimulate internalization during infection with the influenza A virus [57] but also takes part in Epstein-Barr virus- (EBV-) mediated immunosuppression and oncogenesis [58]. Studies on the molecular chaperonin HSP60 confirmed its interactions with Ebola virus infection [59] and human hepatitis B virus polymerase [60], while HSP70 plays a crucial role in mediating the progression of Zika virus in infected host cells [61]. Histamine, on the other hand, contributes to severe pneumonia in pigs infected with the H1N1 influenza virus [62], whereas levels of histamine and leukotrienes presented in acute dengue patients are closely related to disease severity [63].

Our research findings were consistent with previous studies on monkeypox infection as mentioned above; the GO and pathway analyses revealed similar enriched pathways for both monkeypox-infected monkey and human models, including IL-1, IL-33, IL-17, IL-18, NF-*κ*B, MAPKs, and TNF-R2 signaling (Figure 3). However, interestingly, we also discovered relationships between monkeypox and GPCRs, HSP60/70, histamine, plasmin, and histone cluster-related signaling (Supplementary Figures A1, A2) that have rarely been reported before.

Regarding 24 common genes that were highly expressed in both *in vitro* models (Figure 4), when compared to the previous literature, similar expression patterns were also observed in case of CXCL1, TNFAIP3, BIRC3, IL6, CCL2, ZC3H12A, IL11, CSF2, LIF, PTX3, IER3, EGR1, ADORA2A, DUOX1, and several histone cluster family members such as HIST1H3D and HIST1H2BJ. According to a recent study, there were dramatic increases in serum concentrations of IL1-RA, IL-6, and IFN-*γ* in cynomolgus macaques after being infected with aerosolized MPV [64]. In myeloid cells, TNFAIP3 deficiency helps protect host cells against invasion by the influenza A virus [65]. Both ZC3H12A/MCPIP1 and CDKN1A/p21 may contribute to the synergistic effect on replication control and pharmacological manipulation of HIV-1 [66]. Infection with SARS-CoV-2 and several influenza viruses can lead to alterations in CSF2 and IL-11 expressions [67–69]. PTX3, the expression of which was reported to be upregulated by TNF-*α* during acute lung injury, was recently found to serve as a reliable prognostic indicator in predicting short-term mortality from SARS-CoV-2 [70]. Apoptosis and impacted viral replication were found to be induced by infection with the Venezuelan equine encephalitis virus; however, knockdown of early growth response 1 (EGR1) significantly inhibited these signaling pathways [71]. Two members of the NADPH oxidase (NOX) family, including the DUOX1 and DUOX2 proteins, are potential therapeutic targets against infectious diseases caused by the influenza A virus [72]. Previous research demonstrated that DNA viruses exploit host cellular epigenetic processes to their advantage during infection [73, 74]. Most interestingly, several histone cluster family members were reported to be involved in differential responses of human fetal brain neural stem cells to Zika virus infection [75]. For example, three members of the histone cluster 1 H2B (HIST1H2B) family, namely HIST1H2BB, HIST1H2BK, and HIST1H2BO, were among those hub genes and pathways that contributed to Zika virus infection [76]. However, only a limited number of studies have described such relationships in terms of monkeypox. Interestingly, results of our bioinformatics analysis revealed that expression level of certain histone cluster family members, such as HIST1H3D, HIST1H2BJ, HIST1H2AK, HIST1H2AD, HIST1H2AC, HIST1H1B, HIST2H2AB, HIST1H2BM, HIST1H2BH, and HIST1H2BB, were elevated in both monkeypox-infected cell models of monkey and human. These data suggested that some of these epigenetic regulators may also play important roles in monkeypox infection (Figure 4). Cross-validation performed in the 6-hour monkeypox-infected human immortal epithelial cancer (HeLa) cell model (GSE24125) [77] revealed similar patterns of elevated expressions of the aforementioned genes (Supplementary Figure A6). As the most challenging aspect of diagnosing monkeypox infection is distinguishing monkeypox vesicular rash eruption from those of other diseases [78], our study provides a prospect of the histone cluster family as potential regulators correlated with monkeypox infection. Since the current study only focused on signaling pathways regulated by DEGs caused by monkeypox infection, further investigation into the underlying pathogenesis is required for consolidated guidelines for monkeypox treatment in the future.

## 5. Conclusion

In the current study, we focused on investigating genetic signatures and their related pathways in two different monkeypox-infected models. Our research findings revealed the critical roles of multiple genes and their regulatory pathways in both monkeypox-infected models, which not only directly in line with previous literature but also provided novel functions as well as highly expressed genes related to MPV infection that had been less well revealed in the past. In the context of widespread epidemics of viral infectious diseases that are prevalent today, this work may contribute to bridging the traditional gap between bench research and clinical applications. However, with attempts to validate and examine the potentials of these genes as novel antiviral therapies, further expanding research on disease basics may provide more-concrete perspectives.

## Data Availability

The datasets used and analyzed during the current study are available from the corresponding authors on reasonable request.

## Supplementary Materials

Supplementary Materials Figure S1: MetaCore-generated pathways regulated by top upregulated genes in the monkeypox-infected *Macaca mulatta* kidney epithelial (MK2) cell model. Figure S2: MetaCore-generated Endogenous Metabolic Network among top upregulated genes in the monkeypox-infected *Macaca mulatta* kidney epithelial (MK2) cells, performed on the GSE21001 dataset. Figure S3: MetaCore-generated pathways related to top upregulated genes in the human immortal epithelial cancer (HeLa) cell model. Figure S4: MetaCore-generated Endogenous Metabolic Network among top upregulated genes in the human immortal epithelial cancer (HeLa) cell model, performed on the GSE36854 dataset. Figure S5: Kyoto Encyclopedia of Genes and Genomes (KEGG) enrichment pathways in Formula A analysis in different models. Figure S6: heatmap displaying specific differentially regulated genes within the monkeypox-infected human immortal epithelial cancer (HeLa) cell model (GSE24125) versus the mock-infected group. Table S1: top 50 upregulated pathways in the MetaCore analysis of potential maps. Comparison between monkeypox-infected *Macaca mulatta* kidney epithelial (MK2) cells and human epithelial (HeLa) cells from the GSE21001 and GSE36854 datasets. Table S2: top 50 upregulated pathways in the MetaCore analysis of potential Endogenous Metabolic Networks. Comparison between monkeypox-infected *Macaca mulatta* kidney epithelial (MK2) cells and human epithelial (HeLa) cells from the GSE21001 and GSE36854 datasets. Table S3: top 50 upregulated pathways in the MetaCore analysis of potential maps. Comparison between monkeypox-infected and mock-infected*Macaca mulatta* kidney epithelial (MK2) cells from the GSE21001 dataset. Table S4: top 50 upregulated pathways in the MetaCore analysis of potential Endogenous Metabolic Networks. Comparison between monkeypox-infected and mock-infected*Macaca mulatta* kidney epithelial (MK2) cells from the GSE21001 dataset. Table S5: top 50 upregulated pathways in the MetaCore analysis of potential maps. Comparison between monkeypox-infected and mock-infected human epithelial (HeLa) cells from the GSE36854 dataset. Table S6: top 50 upregulated pathways in the MetaCore analysis of potential Endogenous Metabolic Networks. Comparison between monkeypox-infected and mock-infected human epithelial (HeLa) cells from the GSE36854 dataset.

## References

[1] Tambo E., Al-Nazawi A. M. (2022). Combating the global spread of poverty-related monkeypox outbreaks and beyond. *Infectious Diseases of Poverty*.

[2] Essbauer S., Pfeffer M., Meyer H. (2010). Zoonotic poxviruses. *Veterinary Microbiology*.

[3] Shchelkunov S. N., Totmenin A. V., Babkin I. V. (2001). Human monkeypox and smallpox viruses: genomic comparison. *Human monkeypox and smallpox viruses: genomic comparison*.

[4] Parker S., Schultz D. A., Meyer H., Buller R. M. (2014). Smallpox and Monkeypox Viruses☆. *in Reference Module in Biomedical Sciences*.

[5] Di Giulio D. B., Eckburg P. B. (2004). Human monkeypox: an emerging zoonosis. *The Lancet Infectious Diseases*.

[6] Kozlov M. (2022). Monkeypox goes global: why scientists are on alert. *Nature*.

[7] Breman J. G., Kalisa R., Steniowski M. V., Zanotto E., Gromyko A. I., Arita I. (1980). Human monkeypox, 1970-79. *Bulletin of the World Health Organization*.

[8] Meyer H., Perrichot M., Stemmler M. (2002). Outbreaks of disease suspected of being due to human monkeypox virus infection in the Democratic Republic of Congo in 2001. *Journal of Clinical Microbiology*.

[9] Nguyen P. Y., Ajisegiri W. S., Costantino V., Chughtai A. A., MacIntyre C. R. (2021). Reemergence of human monkeypox and declining population immunity in the context of urbanization, Nigeria, 2017-2020. *Emerging Infectious Diseases*.

[10] Vivancos R., Anderson C., Blomquist P. (2022). Community transmission of monkeypox in the United Kingdom, April to May 2022. *Eurosurveillance*.

[11] Titanji B., Tegomoh B., Nematollahi S., Konomos M., Kulkarni P. A. (2022). Monkeypox-a contemporary review for healthcare professionals. *in Open Forum Infectious Diseases*.

[12] Kumar N., Acharya A., Gendelman H. E., Byrareddy S. N. (2022). The 2022 outbreak and the pathobiology of the monkeypox virus. *Journal of Autoimmunity*.

[13] Heskin J., Belfield A., Milne C. (2022). Transmission of monkeypox virus through sexual contact - a novel route of infection. *Journal of Infection*.

[14] Petersen E., Zumla A., Hui D. (2022). Vaccination for monkeypox prevention in persons with high-risk sexual behaviours to control on-going outbreak of monkeypox virus clade 3. *International Journal of Infectious Diseases*.

[15] W H Organization (2022). Surveillance, case investigation and contact tracing for monkeypox: interim guidance, 22 May 2022. *World Health Organization*.

[16] Dashraath P., Nielsen-Saines K., Mattar C., Musso D., Tambyah P., Baud D. (2022). Guidelines for pregnant individuals with monkeypox virus exposure. *The Lancet*.

[17] Kugelman J. R., Johnston S. C., Mulembakani P. M. (2014). Genomic variability of monkeypox virus among humans, Democratic Republic of the Congo. *Emerging Infectious Diseases*.

[18] Awan U. A., Riasat S., Naeem W., Kamran S., Khattak A. A., Khan S. (2022). Monkeypox: a new threat at our doorstep!. *The Journal of Infection*.

[19] Alkhalil A., Hammamieh R., Hardick J., Ichou M. A., Jett M., Ibrahim S. (2010). Gene expression profiling of monkeypox virus-infected cells reveals novel interfaces for host-virus interactions. *Virology Journal*.

[20] Bourquain D., Dabrowski P. W., Nitsche A. (2013). Comparison of host cell gene expression in cowpox, monkeypox or vaccinia virus-infected cells reveals virus-specific regulation of immune response genes. *Virology Journal*.

[21] Wang C.-Y., Chang Y.-C., Kuo Y.-L. (2019). Mutation of the PTCH1 gene predicts recurrence of breast cancer. *Scientific reports*.

[22] Cooke D. L., McCoy D. B., Halbach V. V. (2018). Endovascular biopsy: in vivo cerebral aneurysm endothelial cell sampling and gene expression analysis. *Translational Stroke Research*.

[23] Weng T. Y., Wu H. F., Li C. Y. (2018). Homoharringtonine induced immune alteration for an efficient anti-tumor response in mouse models of non-small cell lung adenocarcinoma expressing Kras mutation. *Scientific Reports*.

[24] Shahi P., Wang C. Y., Lawson D. A. (2017). ZNF503/Zpo2drives aggressive breast cancer progression by down-regulation of GATA3 expression. *Proceedings of the National Academy of Sciences of the United States of America*.

[25] Phan N. N., Wang C. Y., Lin Y. C. (2014). The novel regulations of MEF2A, CAMKK2, CALM3, and TNNI3 in ventricular hypertrophy induced by arsenic exposure in rats. *Toxicology*.

[26] Hung Y. H., Chan Y. S., Chang Y. S. (2014). Fatty acid metabolic enzyme acyl-CoA thioesterase 8 promotes the development of hepatocellular carcinoma. *Oncology Reports*.

[27] Da W. H., Sherman B. T., Lempicki R. A. (2009). Systematic and integrative analysis of large gene lists using DAVID bioinformatics resources. *Nature Protocols*.

[28] Da W. H., Sherman B. T., Lempicki R. A. (2009). Bioinformatics enrichment tools: paths toward the comprehensive functional analysis of large gene lists. *Nucleic Acids Research*.

[29] Subramanian A., Tamayo P., Mootha V. K. (2005). Gene set enrichment analysis: a knowledge-based approach for interpreting genome-wide expression profiles. *Proceedings of the National Academy of Sciences of the United States of America*.

[30] Wang C. Y., Chiao C. C., Phan N. N. (2020). Gene signatures and potential therapeutic targets of amino acid metabolism in estrogen receptor-positive breast cancer. *American Journal of Cancer Research*.

[31] Kao T. J., Wu C. C., Phan N. N. (2021). Prognoses and genomic analyses of proteasome 26S subunit, ATPase (PSMC) family genes in clinical breast cancer. *Aging (Albany NY)*.

[32] Wang C. Y., Chao Y. J., Chen Y. L. (2021). Upregulation of peroxisome proliferator-activated receptor-*α* and the lipid metabolism pathway promotes carcinogenesis of ampullary cancer. *International Journal of Medical Sciences*.

[33] Liu H. L., Yeh I. J., Phan N. N. (2020). Gene signatures of SARS-CoV/SARS-CoV-2-infected ferret lungs in short- and long-term models. *Infection, Genetics and Evolution*.

[34] Wu Y. H., Yeh I. J., Phan N. N. (2021). Gene signatures and potential therapeutic targets of Middle East respiratory syndrome coronavirus (MERS-CoV)-infected human lung adenocarcinoma epithelial cells. *Journal of Microbiology, Immunology, and Infection*.

[35] Ashburner M., Ball C. A., Blake J. A. (2000). Gene Ontology: tool for the unification of biology. *Nature Genetics*.

[36] Szklarczyk D., Gable A. L., Nastou K. C. (2021). The STRING database in 2021: customizable protein-protein networks, and functional characterization of user-uploaded gene/measurement sets. *Nucleic Acids Research*.

[37] Wang Y., Luo Y., Yao Y. (2020). Silencing the lncRNA Maclpil in pro-inflammatory macrophages attenuates acute experimental ischemic stroke via LCP1 in mice. *Journal of Cerebral Blood Flow and Metabolism*.

[38] Wang Y., Liu C., Chen Y. (2022). Systemically silencing long non-coding RNAs Maclpil with short interfering RNA nanoparticles alleviates experimental ischemic stroke by promoting macrophage apoptosis and anti-inflammatory activation. *Front Cardiovasc Med*.

[39] Rajeev R., Prathiviraj R., Kiran G. S., Selvin J. (2020). Zoonotic evolution and implications of microbiome in viral transmission and infection. *Virus Research*.

[40] Chao C. M., Lai C. C., Yu W. L. (2022). COVID-19 associated mucormycosis - an emerging threat. *Journal of Microbiology, Immunology, and Infection*.

[41] Fan C. K., Liao C. W., Cheng Y. C. (2013). Factors affecting disease manifestation of toxocarosis in humans: genetics and environment. *Veterinary Parasitology*.

[42] Bai G. H., Lin S. C., Hsu Y. H., Chen S. Y. (2022). The human virome: viral metagenomics, relations with human diseases, and therapeutic applications. *Viruses*.

[43] Lan G. Y., Lee Y. J., Wu J. C. (2022). Serial quantitative chest computed tomography imaging as prognosticators of coronavirus disease 2019 pneumonia. *Journal of the Formosan Medical Association*.

[44] Mwale P. F., Lee C. H., Lin L. T. (2020). Expression, purification, and characterization of anti-zika virus envelope protein: polyclonal and chicken-derived single chain variable fragment antibodies. *International Journal of Molecular Sciences*.

[45] Ye Z. W., Yuan S., Yuen K. S., Fung S. Y., Chan C. P., Jin D. Y. (2020). Zoonotic origins of human coronaviruses. *International Journal of Biological Sciences*.

[46] Zahradník J., Marciano S., Shemesh M. (2021). SARS-CoV-2 variant prediction and antiviral drug design are enabled by RBD in vitro evolution. *Nature Microbiology*.

[47] Xu B., Li G., Guo J. (2021). Angiotensin-converting enzyme 2, coronavirus disease 2019, and abdominal aortic aneurysms. *Journal of Vascular Surgery*.

[48] Liu C. H., Lu C. H., Lin L. T. (2022). Pandemic strategies with computational and structural biology against COVID-19: a retrospective. *Computational and Structural Biotechnology Journal*.

[49] Lim H. G., Hsiao S. H., Fann Y. C., Lee Y. G. (2022). Robust mutation profiling of SARS-CoV-2 variants from multiple raw illumina sequencing data with cloud workflow. *Genes (Basel)*.

[50] Yu W. L., Toh H. S., Liao C. T., Chang W. T. (2021). A double-edged sword-cardiovascular concerns of potential anti-COVID-19 drugs. *Cardiovascular Drugs and Therapy*.

[51] Poly T. N., Islam M. M., Li Y. J. (2021). Application of artificial intelligence for screening COVID-19 patients using digital images: meta-analysis. *JMIR Medical Informatics*.

[52] Yu C. S., Chang S. S., Chang T. H. (2021). A COVID-19 pandemic artificial intelligence-based system with deep learning forecasting and automatic statistical data acquisition: development and implementation study. *Journal of Medical Internet Research*.

[53] Zhang H., Chen T., Zhang Y. (2022). Crucial genes in aortic dissection identified by weighted gene coexpression network analysis. *Journal of Immunology Research*.

[54] Li Y., Wang Y., Yao Y. (2020). Systematic study of the immune components after ischemic stroke using CyTOF techniques. *Journal of Immunology Research*.

[55] Ruby J., Bluethmann H., Peschon J. J. (1997). Antiviral activity of tumor necrosis factor (TNF) is mediated via p55 and p75 TNF receptors. *The Journal of Experimental Medicine*.

[56] Orzalli M. H., Smith A., Jurado K. A., Iwasaki A., Garlick J. A., Kagan J. C. (2018). An antiviral branch of the IL-1 signaling pathway restricts immune-evasive virus replication. *Molecular Cell*.

[57] Wang G., Jiang L., Wang J. (2020). The G protein-coupled receptor FFAR2 promotes internalization during influenza a virus entry. *Journal of Virology*.

[58] Tsutsumi N., Qu Q., Mavri M. (2021). Structural basis for the constitutive activity and immunomodulatory properties of the Epstein-Barr virus-encoded G protein-coupled receptor BILF1. *Immunity*.

[59] Fausther-Bovendo H., Qiu X., McCorrister S. (2017). Ebola virus infection induces autoimmunity against dsDNA and HSP60. *Scientific Reports*.

[60] Park S. G., Jung G. (2001). Human hepatitis B virus polymerase interacts with the molecular chaperonin Hsp60. *Journal of Virology*.

[61] Pujhari S., Brustolin M., Macias V. M. (2019). Heat shock protein 70 (Hsp70) mediates Zika virus entry, replication, and egress from host cells. *Emerg Microbes Infect*.

[62] Jang Y., Jin M., Seo S. H. (2018). Histamine contributes to severe pneumonia in pigs infected with 2009 pandemic H1N1 influenza virus. *Archives of Virology*.

[63] Silva T., Jeewandara C., Gomes L. (2021). Urinary leukotrienes and histamine in patients with varying severity of acute dengue. *PLoS One*.

[64] Tree J. A., Hall G., Pearson G. (2015). Sequence of pathogenic events in cynomolgus macaques infected with aerosolized monkeypox virus. *Journal of Virology*.

[65] Maelfait J., Roose K., Bogaert P. (2012). A20 (Tnfaip3) deficiency in myeloid cells protects against influenza A virus infection. *PLoS Pathogens*.

[66] de Azevedo S. S. D., Ribeiro-Alves M., Côrtes F. H. (2020). Increased expression of CDKN1A/p21 in HIV-1 controllers is correlated with upregulation of ZC3H12A/MCPIP1. *Retrovirology*.

[67] Chandrashekar D. S., Athar M., Manne U., Varambally S. (2021). Comparative transcriptome analyses reveal genes associated with SARS-CoV-2 infection of human lung epithelial cells. *Scientific Reports*.

[68] Cheng L. C., Kao T. J., Phan N. N. (2021). Novel signaling pathways regulate SARS-CoV and SARS-CoV-2 infectious disease. *Medicine (Baltimore)*.

[69] Wu Y. H., Yeh I. J., Phan N. N. (2021). Severe acute respiratory syndrome coronavirus (SARS-CoV)-2 infection induces dysregulation of immunity: in silico gene expression analysis. *International Journal of Medical Sciences*.

[70] Brunetta E., Folci M., Bottazzi B. (2021). Macrophage expression and prognostic significance of the long pentraxin PTX3 in COVID-19. *Nature Immunology*.

[71] Dahal B., Lin S. C., Carey B. D. (2020). EGR1 upregulation following Venezuelan equine encephalitis virus infection is regulated by ERK and PERK pathways contributing to cell death. *Virology*.

[72] Vlahos R., Selemidis S. (2014). NADPH oxidases as novel pharmacologic targets against influenza A virus infection. *Molecular Pharmacology*.

[73] Saud Z., Hitchings M. D., Butt T. M. (2021). Nanopore sequencing and de novo assembly of a misidentified camelpox vaccine reveals putative epigenetic modifications and alternate protein signal peptides. *Scientific Reports*.

[74] Knipe D. M. (2015). Nuclear sensing of viral DNA, epigenetic regulation of herpes simplex virus infection, and innate immunity. *Virology*.

[75] McGrath E. L., Rossi S. L., Gao J. (2017). Differential responses of human fetal brain neural stem cells to Zika virus infection. *Stem Cell Reports*.

[76] Brahma R., Gurumayum S., Naorem L. D., Muthaiyan M., Gopal J., Venkatesan A. (2018). Identification of hub genes and pathways in Zika virus infection using RNA-Seq data: a network-based computational approach. *Viral Immunology*.

[77] Rubins K. H., Hensley L. E., Relman D. A., Brown P. O. (2011). Stunned silence: gene expression programs in human cells infected with monkeypox or vaccinia virus. *PLoS One*.

[78] Jezek Z., Szczeniowski M., Paluku K. M., Mutombo M., Grab B. (1988). Human monkeypox: confusion with chickenpox. *Acta Tropica*.

