# Supplementary material for: Comparison of Transcriptomic Signatures between Monkeypox-Infected Monkey and Human Cell Lines
Source: J Immunol Res. 2022 Sep 1;2022:3883822. doi: 10.1155/2022/3883822 (PMC9458371; doi:10.1155/2022/3883822)
Supplement: Supplementary Materials — Figure S1: MetaCore-generated pathways regulated by top upregulated genes in the monkeypox-infected Macaca mulatta kidney epithelial (MK2) cell model. Figure S2: MetaCore-generated Endogenous Metabolic Network among top upregulated genes in the monkeypox-infected Macaca mulatta kidney epithelial (MK2) cells, performed on the GSE21001 dataset. Figure S3: MetaCore-generated pathways related to top upregulated genes in the human immortal epithelial cancer (HeLa) cell model. Figure S4: MetaCore-generated Endogenous Metabolic Network among top upregulated genes in the human immortal epithelial cancer (HeLa) cell model, performed on the GSE36854 dataset. Figure S5: Kyoto Encyclopedia of Genes and Genomes (KEGG) enrichment pathways in Formula A analysis in different models. Figure S6: heatmap displaying specific differentially regulated genes within the monkeypox-infected human immortal epithelial cancer (HeLa) cell model (GSE24125) versus the mock-infected group. Table S1: top 50 upregulated pathways in the MetaCore analysis of potential maps. Comparison between monkeypox-infected Macaca mulatta kidney epithelial (MK2) cells and human epithelial (HeLa) cells from the GSE21001 and GSE36854 datasets. Table S2: top 50 upregulated pathways in the MetaCore analysis of potential Endogenous Metabolic Networks. Comparison between monkeypox-infected Macaca mulatta kidney epithelial (MK2) cells and human epithelial (HeLa) cells from the GSE21001 and GSE36854 datasets. Table S3: top 50 upregulated pathways in the MetaCore analysis of potential maps. Comparison between monkeypox-infected and mock-infectedMacaca mulatta kidney epithelial (MK2) cells from the GSE21001 dataset. Table S4: top 50 upregulated pathways in the MetaCore analysis of potential Endogenous Metabolic Networks. Comparison between monkeypox-infected and mock-infectedMacaca mulatta kidney epithelial (MK2) cells from the GSE21001 dataset. Table S5: top 50 upregulated pathways in the MetaCore analysis of potential maps [file 3883822.f1.docx]

**Supplementary Materials**

**
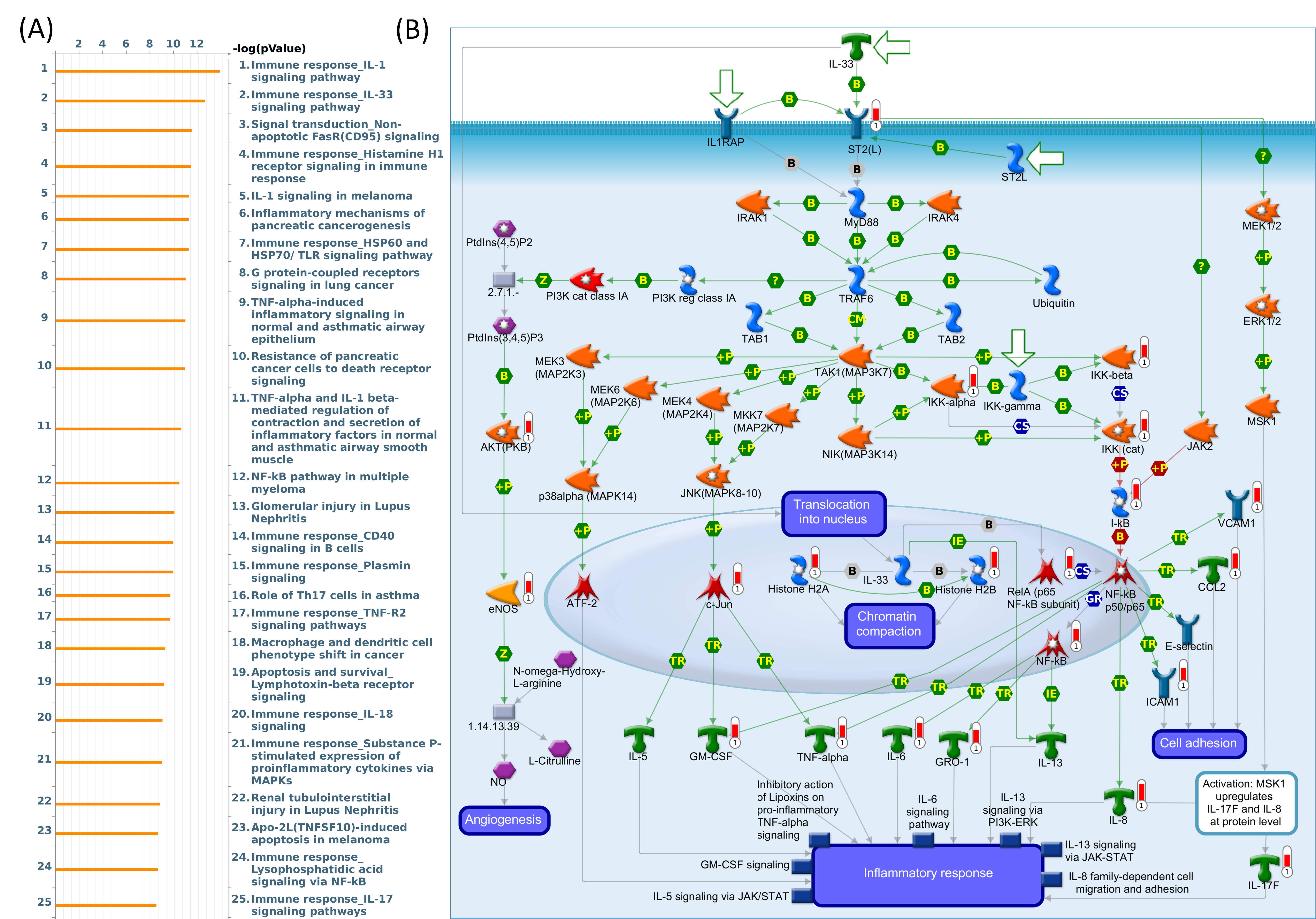
**

**Figure A1: MetaCore-generated pathways regulated by top upregulated genes in the monkeypox-infected Macaca mulatta kidney epithelial (MK2) cell model.** (A) Biological pathways enriched corresponding to genes from the top upregulated genes within the GSE21001 dataset, using MetaCore software. (B) Related pathways and network analyses by MetaCore confirmed the vital role of the “Immune response_IL-33 signaling pathway” in the MK2 model.


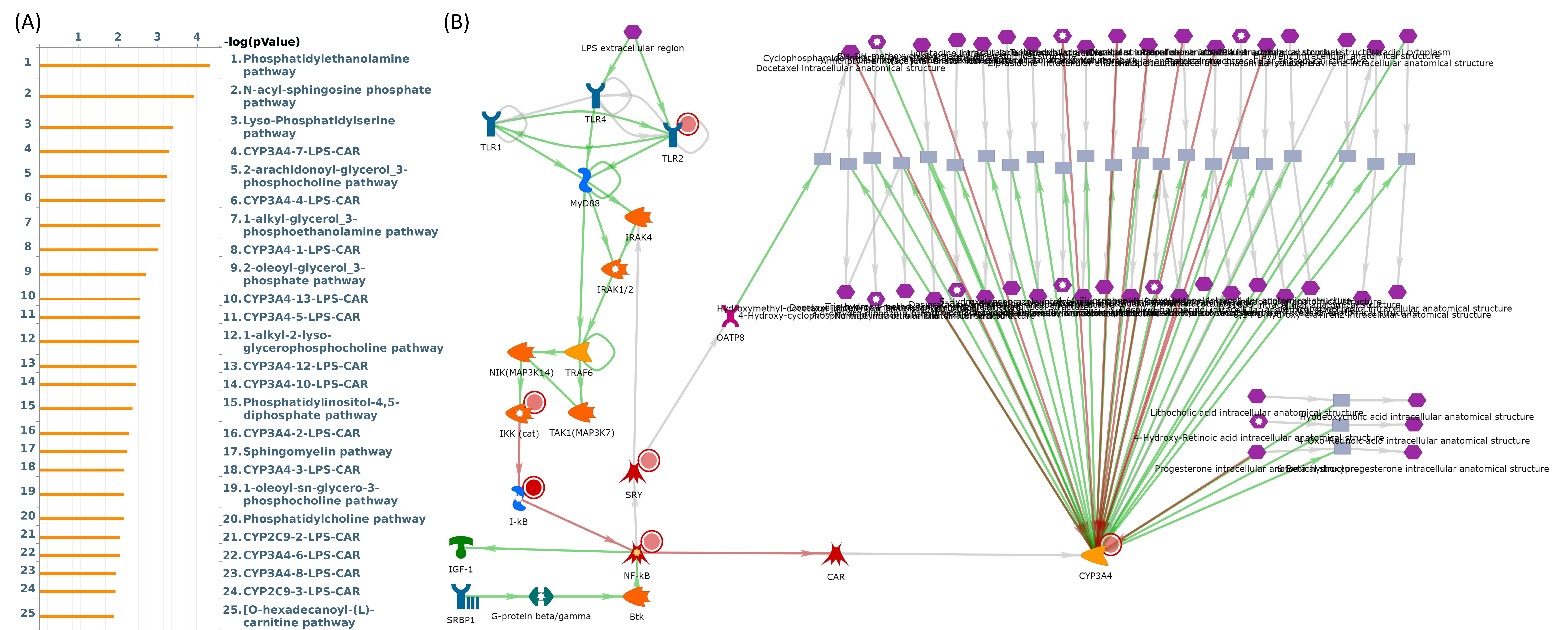
FigureA2. MetaCore-generated Endogenous Metabolic Network among top upregulated genes in the monkeypox-infected *Macaca mulatta* kidney epithelial (MK2) cell , performed on the GSE21001 dataset. (A) Names of enriched metabolic pathways involved in this network, in decreasing order of log(p values). (B) Visualization of the sub-molecular Endogenous Metabolic Network generated by MetaCore, “CYP3A4-7-LPS-CAR” is the downstream pathway that plays a major role in the MK2 model.


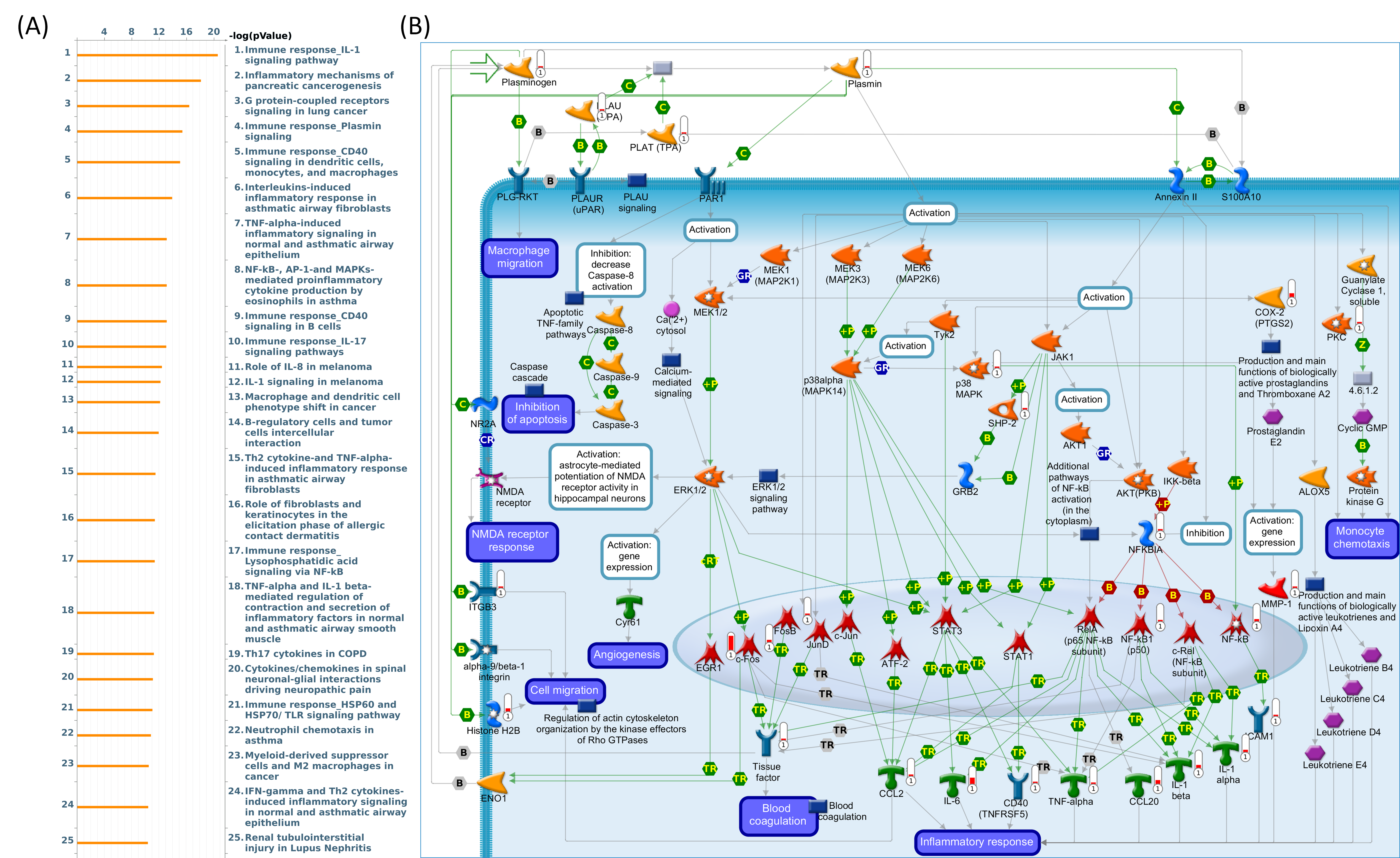


**Figure A3. MetaCore-generated pathways related to top upregulated genes in the human immortal epithelial cancer (HeLa) cell model.** (A) Biological pathways enriched corresponding to genes from the top upregulated genes within the GSE36854 dataset, using MetaCore software. (B) Related pathways and network analyses by MetaCore confirmed the vital role of “Immune response_Plasmin signaling” in the HeLa model.


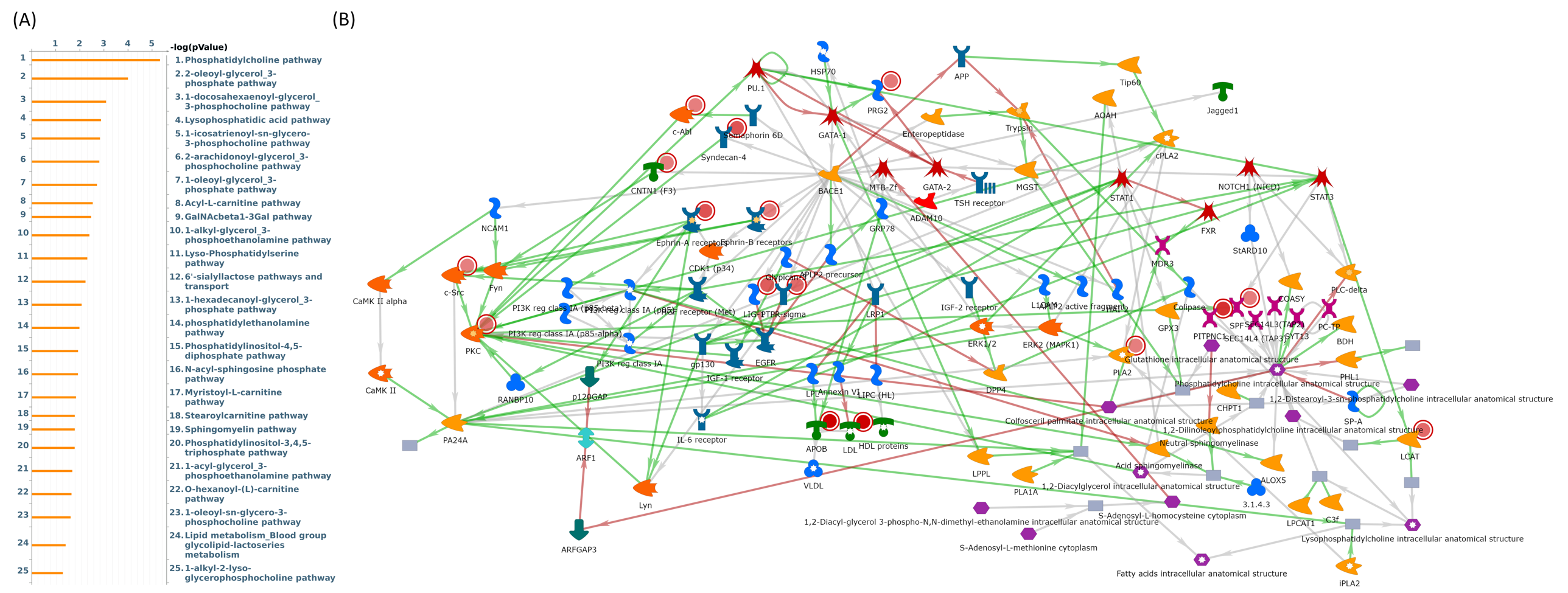
Figure A4. MetaCore-generated Endogenous Metabolic Network among top upregulated genes in the human immortal epithelial cancer (HeLa) cell model, performed on the GSE36854 dataset. (A) Names of enriched metabolic pathways involved in this network, in decreasing order of log(p values). (B) Visualization of the sub-molecular Endogenous Metabolic Network generated by MetaCore, the sphatidylcholine pathway” was the downstream pathway that plays a major role in the HeLa model.

**Figure A5. Kyoto Encyclopedia of Genes and Genomes (KEGG) enrichment pathways analysis in different models.** (A) Top 50 enriched KEGG pathways associated with upregulated genes shared by the monkeypox-infected Macaca mulatta kidney epithelial (MK2) cell model and the human immortal epithelial cancer (HeLa) cell model. (B) The top 30 enriched KEGG pathways associated with upregulated genes within the monkeypox-infected M. mulatta kidney epithelial (MK2) cell model. (C) The top 30 enriched KEGG pathways associated with upregulated genes within the monkeypox-infected human immortal epithelial cancer (HeLa) cell model. The lengths of the horizontal bars corresponding to each pathway indicate the fold enrichment values, with specific color and dot sizes representing false discovery rates (FDRs) and the numbers of genes involved, respectively.

**
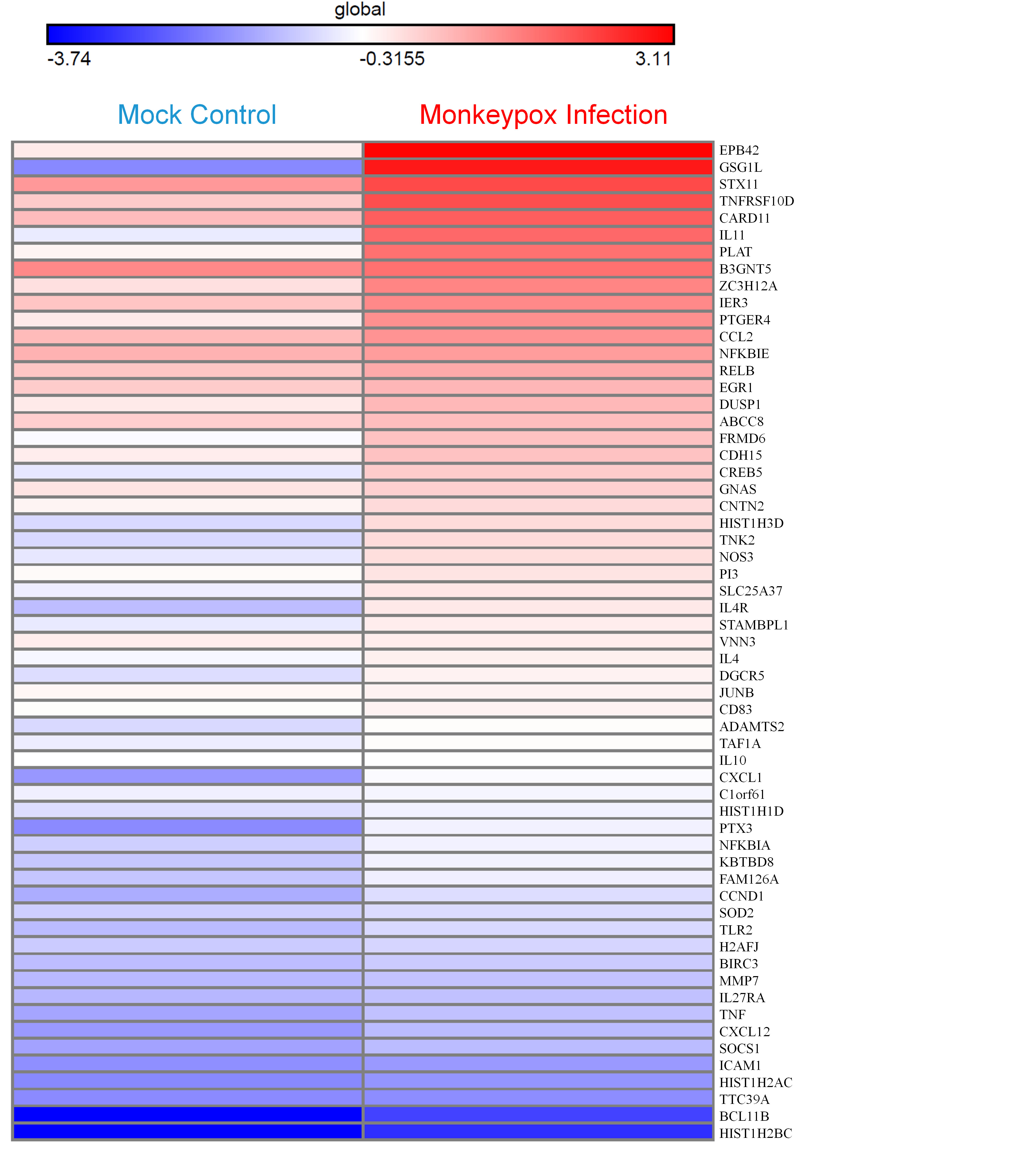
Figure A6. Heatmap displaying specific differentially regulated genes within the monkeypox-infected human immortal epithelial cancer (HeLa) cell model (GSE24125) versus the mock control group.** The gene list was retrieved from common upregulated genes between the monkeypox-infected Macaca mulatta kidney epithelial (MK2) cell model (GSE21001) and the human immortal epithelial cancer (HeLa) cell model (GSE36854). Fold changes of transcriptomic data are displayed as gradient colors ranked from blue to red, representing low to high expression levels of the given genes.

Table A1. Top 50 upregulated pathways in the MetaCore analysis of potential maps. Comparison between monkeypox-infected *Macaca mulatta* kidney epithelial (MK2) cells and human epithelial (HeLa) cells from the GSE21001 and GSE36854 datasets

| # | Maps | pValue | Network Objects from Active Data |
| --- | --- | --- | --- |
| 1 | Immune response_IL-1 signaling pathway | 4.25E-21 | I-kB, NF-kB, EGR1, AP-1, NF-kB2 (p100), ICAM1, GM-CSF, MMP-1, c-IAP2, GRO-1, IL-6, MMP-13, CCL2, MCPIP, NF-kB2 (p52), PLAU (UPA), TNF-alpha, NF-kB p52/RelB |
| 2 | IL-1 signaling in melanoma | 3.25E-14 | I-kB, NF-kB, GRO-3, AP-1, ICAM1, GRO-2, MMP-1, GRO-1, IL-6, SOD2, CCL2 |
| 3 | NF-kB pathway in multiple myeloma | 1.89E-13 | I-kB, NF-kB, NF-kB2 (p100), c-IAP1, c-IAP2, IL-6, NF-kB2 (p52), Cyclin D1, CD40(TNFRSF5), NF-kB p52/RelB |
| 4 | Immune response_CD40 signaling in B cells | 2.42E-13 | I-kB, NF-kB, AP-1, NF-kB2 (p100), ICAM1, c-IAP1, c-IAP2, IL-6, NFKBIA, NF-kB2 (p52), CD40(TNFRSF5), NF-kB p52/RelB, BFL1 |
| 5 | TNF-alpha-induced inflammatory signaling in normal and asthmatic airway epithelium | 4.77E-13 | I-kB, NF-kB, IL-4, ICAM1, GM-CSF, GRO-1, IL-6, CCL2, NFKBIA, TNF-alpha |
| 6 | Immune response_Plasmin signaling | 5.1E-13 | NF-kB, EGR1, ICAM1, MMP-1, IL-6, CCL2, Histone H2B, NFKBIA, PLAT (TPA), PLAU (UPA), TNF-alpha, CD40(TNFRSF5) |
| 7 | Glomerular injury in Lupus Nephritis | 6.97E-13 | NF-kB, GRO-3, A20, ICAM1, GM-CSF, GRO-2, MMP-1, GRO-1, IL-6, CCL2, CSF1, Cyclin D1, TNF-alpha |
| 8 | Immune response_IL-33 signaling pathway | 1.55E-12 | I-kB, NF-kB, Histone H2A, ICAM1, GM-CSF, GRO-1, IL-6, CCL2, eNOS, Histone H2B, TNF-alpha |
| 9 | Macrophage and dendritic cell phenotype shift in cancer | 2.1E-12 | I-kB, NF-kB, TLR2, IL-4, GM-CSF, IRE1, IL-6, CSF1, SOCS1, IL-10, PGE2R4, TNF-alpha, CD40(TNFRSF5) |
| 10 | Renal tubulointerstitial injury in Lupus Nephritis | 5.79E-12 | I-kB, ICAM1, GM-CSF, IL-6, CCL2, CSF1, SOCS1, PLAU (UPA), TNF-alpha, CD40(TNFRSF5), NF-kB p52/RelB |
| 11 | Signal transduction_Non-apoptotic FasR(CD95) signaling | 6.55E-12 | I-kB, NF-kB, GRO-3, EGR1, AP-1, GRO-2, GRO-1, IL-6, CCL2, eNOS, NFKBIA, TNF-alpha |
| 12 | Inflammatory mechanisms of pancreatic cancerogenesis | 8.2E-12 | NF-kB, AP-1, ICAM1, GRO-1, IL-6, CCL2, SDF-1, NFKBIA, PLAU (UPA), TNF-alpha, CD40(TNFRSF5) |
| 13 | Th2 cytokine- and TNF-alpha-induced inflammatory response in asthmatic airway fibroblasts | 9.31E-12 | NF-kB, IL-4, ICAM1, GM-CSF, IL-6, IL4RA, CCL2, TNF-alpha, CD40(TNFRSF5) |
| 14 | Signal transduction_NF-kB activation pathways | 1.18E-11 | I-kB, NF-kB, RelB (NF-kB subunit), TLR2, NF-kB2 (p100), c-IAP1, c-IAP2, NF-kB2 (p52), TNF-alpha, NF-kB p52/RelB |
| 15 | Immune response_HSP60 and HSP70/ TLR signaling pathway | 2.16E-11 | I-kB, NF-kB, CD83, AP-1, TLR2, ICAM1, IL-6, IL-10, TNF-alpha, CD40(TNFRSF5) |
| 16 | Immune response_CD40 signaling in dendritic cells, monocytes, and macrophages | 4.59E-11 | NF-kB, CD83, NF-kB2 (p100), c-IAP2, IL-6, NFKBIA, NF-kB2 (p52), IL-10, TNF-alpha, CD40(TNFRSF5), NF-kB p52/RelB |
| 17 | Glucocorticoid- and LABA-mediated inhibition of pro-inflammatory signaling in airway fibroblasts/myofibroblasts | 5.46E-11 | NF-kB, AP-1, G-protein alpha-s, IL-4, ICAM1, GM-CSF, IL-6, TNF-alpha |
| 18 | Apoptosis and survival_Anti-apoptotic TNFs/NF-kB/Bcl-2 pathway | 5.6E-11 | I-kB, NF-kB, RelB (NF-kB subunit), NF-kB2 (p100), NF-kB2 (p52), TNF-alpha, CD40(TNFRSF5), NF-kB p52/RelB, BFL1 |
| 19 | Release of pro-inflammatory factors and proteases by alveolar macrophages in asthma | 8.78E-11 | NF-kB, TLR2, GM-CSF, GRO-2, MMP-1, GRO-1, IL-6, CCL2, TNF-alpha |
| 20 | Immune response_TNF-R2 signaling pathways | 1.09E-10 | I-kB, NF-kB, AP-1, NF-kB2 (p100), c-IAP1, c-IAP2, NF-kB2 (p52), TNF-alpha, NF-kB p52/RelB |
| 21 | TNF-alpha and IL-1 beta-mediated regulation of contraction and secretion of inflammatory factors in normal and asthmatic airway smooth muscle | 1.5E-10 | NF-kB, GRO-3, GM-CSF, GRO-2, GRO-1, IL-6, CCL2, Histone H3, NFKBIA, TNF-alpha |
| 22 | Immune response_Histamine H1 receptor signaling in immune response | 1.65E-10 | I-kB, ICAM1, GM-CSF, MMP-1, IL-6, MMP-13, eNOS, NFKBIA, TNF-alpha |
| 23 | B-regulatory cells and tumor cells intercellular interaction | 2.04E-10 | NF-kB, IL-4, GM-CSF, IL-6, IL4RA, CCL2, IL27RA, IL-10, TNF-alpha, CD40(TNFRSF5) |
| 24 | Activation of TNF-alpha-dependent pro-tumoral effect in colorectal cancer | 2.46E-10 | I-kB, NF-kB, G-protein alpha-s, ICAM1, c-IAP2, GRO-1, IL-6, CCL2, TNF-alpha |
| 25 | Interleukins-induced inflammatory response in asthmatic airway fibroblasts | 3.93E-10 | NF-kB, IL-11, ICAM1, GM-CSF, GRO-2, GRO-1, IL-6, CCL2 |
| 26 | Immune response_HMGB1/RAGE signaling pathway | 5.15E-10 | I-kB, NF-kB, TLR2, ICAM1, IL-6, NFKBIA, PLAT (TPA), TNF-alpha, RAGE |
| 27 | Signal transduction_S1P2 receptor activation signaling | 1.49E-09 | NF-kB, EGR1, AP-1, HB-EGF, ICAM1, GRO-1, LIF, CCL2, NFKBIA, Cyclin D1, TNF-alpha |
| 28 | NF-kB-, AP-1- and MAPKs-mediated proinflammatory cytokine production by eosinophils in asthma | 2.29E-09 | NF-kB, AP-1, IL-4, GM-CSF, GRO-1, IL-6, CCL2, TNF-alpha |
| 29 | Vascular endothelial cell damage in SLE | 2.57E-09 | NF-kB, ICAM1, GM-CSF, IL-6, CCL2, CSF1, IL-10, TNF-alpha, RAGE |
| 30 | Release of pro-inflammatory mediators and elastolytic enzymes by alveolar macrophages in COPD | 2.61E-09 | Matrilysin (MMP-7), TLR2, GM-CSF, MMP-1, IL-6, CCL2, TNF-alpha |
| 31 | Role of Th17 cells in asthma | 2.61E-09 | NF-kB, GRO-3, IL-11, HB-EGF, ICAM1, GRO-2, GRO-1 |
| 32 | Myeloid-derived suppressor cells and M2 macrophages in cancer | 2.96E-09 | NF-kB, IL-4, GM-CSF, IL-6, IL4RA, CSF1, IL-10, PGE2R4, RAGE |
| 33 | Cigarette smoke-mediated attenuation of antibacterial and antivirus immune response | 3.42E-09 | AP-1, ICAM1, GM-CSF, IL-6, NFKBIA, TNF-alpha, sICAM1 |
| 34 | Immune response_IL-11 signaling pathway via MEK/ERK and PI3K/AKT cascades | 4.51E-09 | I-kB, IL-11, IL-4, ICAM1, IL-6, NFKBIA, IL-10, Cyclin D1, TNF-alpha |
| 35 | Immune response_IL-3 signaling via JAK/STAT, p38, JNK and NF-kB | 5.55E-09 | I-kB, NF-kB, IL-4, ICAM1, MKP-1, IRE1, IL-6, SOCS1, Cyclin D1, CD40(TNFRSF5) |
| 36 | Autocrine production of eosinophil pro-survival cytokines in asthma | 5.68E-09 | NF-kB, IL-4, ICAM1, GM-CSF, TNF-alpha, CD40(TNFRSF5), Fc alpha receptor |
| 37 | Immune response_OX40L/ OX40 signaling pathway | 5.89E-09 | I-kB, NF-kB, NF-kB2 (p100), IL-4, NF-kB2 (p52), CARD11, IL-10, NF-kB p52/RelB, BFL1 |
| 38 | Pro-inflammatory cytokine release from eosinophils in asthma | 1.14E-08 | IL-4, GM-CSF, IL-6, LIF, TrkA, TNF-alpha, Fc alpha receptor |
| 39 | Inflammatory response in ischemia-reperfusion injury during myocardial infarction | 1.56E-08 | NF-kB, TLR2, ICAM1, IL-6, CCL2, TNF-alpha |
| 40 | Cigarette smoke-induced inflammatory signaling in airway epithelial cells | 1.74E-08 | HB-EGF, ICAM1, GM-CSF, IL-6, NFKBIA, TNF-alpha, sICAM1 |
| 41 | Immune response_Oncostatin M signaling via MAPK | 2.13E-08 | EGR1, AP-1, MMP-1, MMP-13, CCL2, LDLR, LIFR |
| 42 | Expression targets of Tissue factor signaling in cancer | 2.13E-08 | GM-CSF, GRO-1, NFKBIE, CSF1, PLAU (UPA), PTX3 |
| 43 | Role of fibroblasts in the sensitization phase of allergic contact dermatitis | 2.13E-08 | NF-kB, AP-1, IL-6, SDF-1, IL-10, TNF-alpha |
| 44 | Immune response_Th17, Th22 and Th9 cell differentiation | 3.14E-08 | NF-kB, AP-1, IL-4, IL-6, IL-10, TNF-alpha, CD40(TNFRSF5) |
| 45 | Cooperative action of IFN-gamma and TNF-alpha on astrocytes in multiple sclerosis | 3.14E-08 | I-kB, NF-kB, G-protein alpha-s, ICAM1, CCL2, NFKBIA, TNF-alpha |
| 46 | Immune response_IL-17 signaling pathways | 3.59E-08 | I-kB, NF-kB, ICAM1, GM-CSF, MMP-1, GRO-1, IL-6, CCL2 |
| 47 | Immune response_TREM1 signaling pathway | 3.59E-08 | I-kB, TLR2, WBSCR5(NTAL), GM-CSF, IL-6, CCL2, NFKBIA, TNF-alpha |
| 48 | IFN-gamma and Th2 cytokines-induced inflammatory signaling in normal and asthmatic airway epithelium | 3.78E-08 | NF-kB, IL-4, ICAM1, IL-6, IL4RA, CCL2, SOCS1 |
| 49 | Maturation and migration of dendritic cells in skin sensitization | 4.53E-08 | I-kB, NF-kB, CD83, ICAM1, IL-6, TNF-alpha, CD40(TNFRSF5) |
| 50 | Apoptosis and survival_Granzyme A signaling | 4.53E-08 | TLR2, IL-6, CCL2, Histone H3, Histone H1, Histone H2B, TNF-alpha |

**Table A2. Top 50 upregulated pathways in the MetaCore analysis of potential Endogenous Metabolic Networks. Comparison between monkeypox-infected Macaca mulatta kidney epithelial (MK2) cells and human epithelial (HeLa) cells from the GSE21001 and GSE36854 datasets.**

| Enrichment by Metabolic Networks (Endogenous) | | | |
| --- | --- | --- | --- |
| # | Networks | pValue | Network Objects from Active Data |
| 1 | N-acyl-sphingosine phosphate pathway | 8.75E-04 | MMP-1, Galpha(q)-specific peptide GPCRs, eNOS, PLAT (TPA), PLAU (UPA) |
| 2 | 2-arachidonoyl-glycerol_3-phosphocholine pathway | 2.70E-03 | SUR, G-protein alpha-s, LCAT, SUR1, RAGE |
| 3 | 1-icosatrienoyl-sn-glycero-3-phosphocholine pathway | 1.06E-02 | SUR, G-protein alpha-s, LCAT, SUR1 |
| 4 | 1-alkyl-glycerol_3-phosphoethanolamine pathway | 1.54E-02 | ICAM1, CCL2 |
| 5 | phosphatidylethanolamine pathway | 2.52E-02 | LDLR, PLAT (TPA), PLAU (UPA) |
| 6 | (L)-valine pathways and transport | 3.06E-02 | AP-1, ICAM1, SLC7A8 |
| 7 | Carbohydrate metabolism_TCA and tricarboxylic acid transport | 3.40E-02 | Pyruvate kinase, NRSF, KPYR |
| 8 | 1-oleoyl-sn-glycero-3-phosphocholine pathway | 5.34E-02 | G-protein alpha-s, LCAT, SUR1 |
| 9 | 1-docosahexaenoyl-glycerol_3-phosphocholine pathway | 6.35E-02 | G-protein alpha-s, LCAT, SUR1 |
| 10 | Vitamin, mediator and cofactor metabolism_Alpha-tocotrienol | 7.72E-02 | AP-1, NPC1L1 |
| 11 | Ceramide pathway | 1.01E-01 | eNOS, TNF-alpha |
| 12 | 1-acyl-glycerol_3-phosphocholine pathway | 1.29E-01 | LCAT, RAGE |
| 13 | GalNAcbeta1-3Gal pathway | 1.33E-01 | LDLR, Galpha(q)-specific peptide GPCRs |
| 14 | Alpha-L-fucosyl-(1-2)-D-galactose pathway | 1.48E-01 | Alpha 1-antitrypsin, PLAU (UPA) |
| 15 | Lactosylceramide pathway | 1.58E-01 | B3GNT5, eNOS |
| 16 | Aminoacid metabolism_Asparagine, Aspartic acid metabolism and transport | 1.88E-01 | NF-kB, eNOS |
| 17 | Glycine pathways and transport | 1.94E-01 | EGR1, SLC7A8 |
| 18 | (L)-phenylalanine pathways and transport | 1.94E-01 | SLC7A8, GCH1 |
| 19 | Methionine pathways and transport | 1.96E-01 | ICAM1, SLC7A8 |
| 20 | Pentose phosphate pathways and transport | 2.14E-01 | AP-1, JunB |
| 21 | Phosphatidylcholine pathway | 2.33E-01 | LCAT, LIG-1 |
| 22 | N-acetyl-D-galactosamine pathway | 2.49E-01 | TLR2 |
| 23 | Vitamin, mediator and cofactor metabolism_Nitric oxide biosynthesis and transport | 3.06E-01 | eNOS |
| 24 | 1-alkyl-2-lyso-glycerophosphocholine pathway | 3.06E-01 | CCL2 |
| 25 | Lipid metabolism_Blood group glycolipid-neo-lactoseries metabolism | 3.18E-01 | SIA10, B3GNT5 |
| 26 | Lipid metabolism_Blood group glycolipid-lactoseries metabolism | 3.64E-01 | B3GNT5 |
| 27 | Phosphatidylinositol-3,4,5-triphosphate pathway | 3.77E-01 | ARHGEF4 |
| 28 | L-arginine pathways and transport | 3.95E-01 | eNOS |
| 29 | [O-hexadecanoyl-(L)-carnitine pathway | 4.00E-01 | G-protein alpha-s |
| 30 | Steroid metabolism_Aldosterone biosynthesis and metabolism | 4.04E-01 | PAX3 |
| 31 | Carbohydrate metabolism_Pyruvate metabolism and transport_new | 4.17E-01 | KPYR |
| 32 | (S)-citrulline pathway | 4.17E-01 | eNOS |
| 33 | 1-palmitoyl-sn-glycero-3-phosphocholine pathway | 4.50E-01 | LCAT |
| 34 | Glucosylceramide pathways and transport | 4.50E-01 | LCAT |
| 35 | Aminoacid metabolism_Tryptophan metabolism and transport | 4.50E-01 | SLC7A8 |
| 36 | (L)-leucine pathways and transport | 4.58E-01 | SLC7A8 |
| 37 | L-citrulline pathway | 4.65E-01 | eNOS |
| 38 | 1-hexadecanoyl-glycerol_3-phosphate pathway | 4.65E-01 | NRSF |
| 39 | Acyl-L-carnitine pathway | 4.69E-01 | G-protein alpha-s |
| 40 | Sucrose pathway | 4.77E-01 | TLR2 |
| 41 | Glycine pathway | 4.77E-01 | EGR1 |
| 42 | 1,2-didocosapentaenoyl-sn-glycerol_3-phosphate pathway | 4.77E-01 | Tubulin alpha |
| 43 | (L)-threonine pathways and transport | 4.84E-01 | SLC7A8 |
| 44 | Phosphatidylinositol-4,5-diphosphate pathway | 4.84E-01 | ICAM1 |
| 45 | 1,2-dioleoyl-sn-glycerol_3-phosphate pathway | 4.88E-01 | Tubulin alpha |
| 46 | Sphingomyelin pathway | 5.03E-01 | TLR2 |
| 47 | 1,2-didocosahexaenoyl-sn-glycerol_3-phosphate pathway | 5.03E-01 | Tubulin alpha |
| 48 | (L)-alanine pathways and transport | 5.14E-01 | SLC7A8 |
| 49 | 1-acyl-glycerol_3-phosphoethanolamine pathway | 5.17E-01 | Alpha 1-antitrypsin |
| 50 | Carbohydrate metabolism_Sucrose metabolism and transport | 5.44E-01 | IL-4 |

| **Table A3. Top 50 up-regulated pathways in the MetaCore analysis of potential maps. Comparison between Monkeypox - infected Macaca mulatta kidney epithelial (MK2) cells and the mock controls from the GSE21001 datasets.**   \| # \| Maps \| pValue \| Network Objects from Active Data \| \| --- \| --- \| --- \| --- \| \| 1 \| Immune response_IL-1 signaling pathway \| 1.22E-14 \| IL-6, I-kB, EGR1, c-IAP2, AP-1, NF-kB2 (p100), IKK (cat), RelA (p65 NF-kB subunit), GRO-1, MMP-13, MCPIP, NF-kB, iNOS, AKT(PKB), IRF1, ICAM1, CD44, CCL2, NF-kB2 (p52), MMP-1, IL-8, MMP-9, GM-CSF, PLAU (UPA), NF-kB p52/RelB, TNF-alpha \| \| 2 \| Immune response_IL-33 signaling pathway \| 2.27E-13 \| IL-6, ST2(L), I-kB, VCAM1, IKK (cat), RelA (p65 NF-kB subunit), GRO-1, NF-kB, AKT(PKB), ICAM1, CCL2, IKK-alpha, IL-8, IL-17F, Histone H2A, GM-CSF, Histone H2B, IKK-beta, c-Jun, eNOS, TNF-alpha \| \| 3 \| Signal transduction_Non-apoptotic FasR(CD95) signaling \| 2.93E-12 \| GRO-2, IL-6, I-kB, EGR1, c-FLIP(Long), AP-1, IKK (cat), GRO-1, NF-kB, c-FLIP, AKT(PKB), NeuroD3, CCL2, SFK, IL-8, GRO-3, MMP-9, NFKBIA, IKK-beta, c-Jun, IP3 receptor, CFLAR p43, eNOS, TNF-alpha \| \| 4 \| Immune response_Histamine H1 receptor signaling in immune response \| 3.9E-12 \| IL-6, I-kB, VCAM1, RelA (p65 NF-kB subunit), MMP-13, iNOS, PLC-beta, ICAM1, MMP-1, IL-8, MMP-9, GM-CSF, NFKBIA, IKK-beta, c-Jun, IP3 receptor, eNOS, TNF-alpha \| \| 5 \| IL-1 signaling in melanoma \| 5.37E-12 \| GRO-2, IL-6, I-kB, AP-1, VCAM1, IKK (cat), GRO-1, NF-kB, iNOS, ICAM1, SOD2, CCL2, MMP-1, IL-8, GRO-3, MMP-9, c-Jun \| \| 6 \| Inflammatory mechanisms of pancreatic cancerogenesis \| 5.73E-12 \| IL-6, AP-1, IKK (cat), RelA (p65 NF-kB subunit), GRO-1, SHH, CD40(TNFRSF5), NF-kB, iNOS, IRF1, ICAM1, CCL2, IKK-alpha, IL-8, MMP-9, SDF-1, NFKBIA, IKK-beta, PLAU (UPA), c-Jun, TNF-alpha \| \| 7 \| Immune response_HSP60 and HSP70/ TLR signaling pathway \| 5.76E-12 \| IL-6, I-kB, CD83, AP-1, E2N(UBC13), IKK (cat), CD40(TNFRSF5), NF-kB, iNOS, CD14, ICAM1, HSP70, IL-10, IKK-alpha, IL-8, IKK-beta, TLR2, c-Jun, TNF-alpha \| \| 8 \| G protein-coupled receptors signaling in lung cancer \| 1.07E-11 \| HB-EGF, Pyk2(FAK2), PGE2R4, I-kB, Galpha(i)-specific peptide GPCRs, Cyclin D1, Amphiregulin, RelA (p65 NF-kB subunit), VIP receptor 2, Galpha(i)-specific cannabis GPSRs, Galpha(q)-specific peptide GPCRs, G-protein alpha-s, AKT(PKB), CD44, IKK-alpha, IL-8, SSTR5, HB-EGF(mature), SDF-1, CNR2, SSTR3, PGE2R1 \| \| 9 \| TNF-alpha-induced inflammatory signaling in normal and asthmatic airway epithelium \| 1.1E-11 \| IL-6, I-kB, GRO-1, CCL17, PP2A catalytic, NF-kB, IL-4, IRF1, ICAM1, CCL2, IL-8, GM-CSF, NFKBIA, IKK-beta, c-Jun, TNF-alpha \| \| 10 \| Resistance of pancreatic cancer cells to death receptor signaling \| 1.27E-11 \| TRUNDD(TNFRSF10D), Casein kinase I epsilon, c-IAP2, DR5(TNFRSF10B), PRKD1, c-FLIP(Short), c-FLIP(Long), RelA (p65 NF-kB subunit), DR4(TNFRSF10A), c-IAP1, tBid, NFKBIA, IKK-beta, Caspase-3, Bid \| \| 11 \| TNF-alpha and IL-1 beta-mediated regulation of contraction and secretion of inflammatory factors in normal and asthmatic airway smooth muscle \| 2.7E-11 \| GRO-2, IL-6, VCAM1, IKK (cat), RelA (p65 NF-kB subunit), GRO-1, NF-kB, Histone H3, AKT(PKB), PLC-beta, CCL2, IL-8, GRO-3, MMP-9, GM-CSF, NFKBIA, Histone H4, c-Jun, p47-phox, TNF-alpha \| \| 12 \| NF-kB pathway in multiple myeloma \| 3.61E-11 \| IL-6, CYLD, I-kB, c-IAP2, c-FLIP(Long), Cyclin D1, NF-kB2 (p100), IKK (cat), CD40(TNFRSF5), NF-kB, NF-kB2 (p52), IKK-alpha, c-IAP1, IKK-beta, NF-kB p52/RelB \| \| 13 \| Glomerular injury in Lupus Nephritis \| 9.82E-11 \| GRO-2, IL-6, Cyclin D1, VCAM1, RelA (p65 NF-kB subunit), GRO-1, NF-kB, iNOS, AKT(PKB), CSF1, Decorin, IRF1, HMGB1, ICAM1, CCL2, MMP-1, IFN-alpha, IL-8, GRO-3, A20, MMP-9, GM-CSF, TNF-alpha \| \| 14 \| Immune response_CD40 signaling in B cells \| 1.21E-10 \| IL-6, BFL1, I-kB, c-IAP2, AP-1, NF-kB2 (p100), IKK (cat), RelA (p65 NF-kB subunit), CD40(TNFRSF5), NF-kB, IGH@, AKT(PKB), IRF1, ICAM1, c-Rel (NF-kB subunit), NF-kB2 (p52), IKK-alpha, c-IAP1, NFKBIA, NF-kB p52/RelB, c-Jun, ALOX5 \| \| 15 \| Immune response_Plasmin signaling \| 1.21E-10 \| IL-6, EGR1, RelA (p65 NF-kB subunit), CD40(TNFRSF5), NF-kB, AKT(PKB), ICAM1, c-Rel (NF-kB subunit), S100A10, CCL2, PLAT (TPA), MMP-1, Histone H2B, NFKBIA, IKK-beta, PLAU (UPA), Caspase-3, c-Jun, ALOX5, TNF-alpha \| \| 16 \| Role of Th17 cells in asthma \| 2.18E-10 \| HB-EGF, GRO-2, VCAM1, GRO-1, NF-kB, ICAM1, IL-8, IL-17F, IL-22, GRO-3, MRLC, IL-11, ALOX5 \| \| 17 \| Immune response_TNF-R2 signaling pathways \| 2.3E-10 \| I-kB, c-IAP2, AP-1, NF-kB2 (p100), IKK (cat), RelA (p65 NF-kB subunit), NF-kB, AKT(PKB), NF-kB2 (p52), IKK-alpha, c-IAP1, SMURF2, IKK-beta, NF-kB p52/RelB, c-Jun, TNF-alpha \| \| 18 \| Macrophage and dendritic cell phenotype shift in cancer \| 5.94E-10 \| IL-6, PGE2R4, I-kB, IKK (cat), RelA (p65 NF-kB subunit), CD40(TNFRSF5), NF-kB, iNOS, IL-4, IRE1, MER, CSF1, Activin A, c-Rel (NF-kB subunit), JMJD3, IL-10, Jagged1, IFN-alpha, GM-CSF, ILT3, TLR2, SOCS1, TNF-alpha \| \| 19 \| Apoptosis and survival_Lymphotoxin-beta receptor signaling \| 7.89E-10 \| I-kB, NF-kB2 (p100), VCAM1, IKK (cat), RelA (p65 NF-kB subunit), RelB (NF-kB subunit), NF-kB2 (p52), IKK-alpha, c-IAP1, IL-8, SDF-1, IKK-beta, NF-kB p52/RelB, Caspase-3, c-Jun \| \| 20 \| Immune response_IL-18 signaling \| 1.04E-09 \| IL-6, I-kB, AP-1, VCAM1, IKK (cat), NF-kB, iNOS, AKT(PKB), ICAM1, CCL2, IKK-alpha, IL-8, MMP-9, IL18RAP, IKK-beta, c-Jun, p47-phox, TNF-alpha \| \| 21 \| Immune response_Substance P-stimulated expression of pro-inflammatory cytokines via MAPKs \| 1.16E-09 \| GRO-2, IL-6, I-kB, IKK (cat), CCL13, AKT(PKB), PLC-beta, CCL2, IKK-alpha, IL-8, GRO-3, IKK-beta, c-Jun, IP3 receptor, TNF-alpha \| \| 22 \| Renal tubulointerstitial injury in Lupus Nephritis \| 1.81E-09 \| IL-6, I-kB, VCAM1, IKK (cat), RelA (p65 NF-kB subunit), CD40(TNFRSF5), CSF1, ICAM1, CD44, CCL2, IKK-alpha, IFN-alpha, IL-8, GM-CSF, PLAU (UPA), NF-kB p52/RelB, SOCS1, TNF-alpha \| \| 23 \| Apo-2L(TNFSF10)-induced apoptosis in melanoma \| 2.39E-09 \| DR5(TNFRSF10B), c-FLIP(Short), c-FLIP(Long), IKK (cat), RelA (p65 NF-kB subunit), NF-kB, IRE1, DR4(TNFRSF10A), Dynamin-2, IFN-alpha, IL-8, tBid, NFKBIA, Caspase-3, Bid \| \| 24 \| Immune response_Lysophosphatidic acid signaling via NF-kB \| 2.66E-09 \| IL-6, CYLD, PKD, VCAM1, IKK (cat), RelA (p65 NF-kB subunit), NF-kB, ICAM1, CCL2, IL-8, GRO-3, A20, MMP-9, NFKBIA, IKK-beta, Caspase-3 \| \| 25 \| Immune response_IL-17 signaling pathways \| 3.49E-09 \| IL-6, I-kB, IKK (cat), GRO-1, NF-kB, iNOS, AKT(PKB), GCP2, ICAM1, CCL2, MMP-1, IL-8, IL-17F, MMP-9, GM-CSF, IKK-beta, c-Jun \| \| 26 \| Cigarette smoke-mediated attenuation of antibacterial and antivirus immune response \| 5.59E-09 \| IL-6, AP-1, IKK (cat), RelA (p65 NF-kB subunit), IRF1, ICAM1, IL-8, GM-CSF, NFKBIA, IKK-beta, TNF-alpha, sICAM1 \| \| 27 \| Th2 cytokine- and TNF-alpha-induced inflammatory response in asthmatic airway fibroblasts \| 6.19E-09 \| IL-6, VCAM1, RelA (p65 NF-kB subunit), CD40(TNFRSF5), NF-kB, IL-4, IL4RA, ICAM1, CCL2, IL-8, GM-CSF, c-Jun, TNF-alpha \| \| 28 \| Immune response_CD40 signaling in dendritic cells, monocytes, and macrophages \| 6.75E-09 \| IL-6, c-IAP2, CD83, NF-kB2 (p100), IKK (cat), CD40(TNFRSF5), NF-kB, iNOS, AKT(PKB), NF-kB2 (p52), IL-10, IKK-alpha, IL-8, N-Ras, NFKBIA, IKK-beta, NF-kB p52/RelB, c-Jun, TNF-alpha \| \| 29 \| Vascular endothelial cell damage in SLE \| 7.92E-09 \| IL-6, VCAM1, RelA (p65 NF-kB subunit), NF-kB, iNOS, CSF1, HMGB1, ICAM1, CCL2, IL-10, IFN-alpha, IL-8, GM-CSF, Caspase-3, AML1 (RUNX1), TNF-alpha, RAGE \| \| 30 \| Apoptosis and survival_Anti-apoptotic TNFs/NF-kB/Bcl-2 pathway \| 8.22E-09 \| BFL1, I-kB, NGFR(TNFRSF16), NF-kB2 (p100), IKK (cat), RelA (p65 NF-kB subunit), CD40(TNFRSF5), NF-kB, RelB (NF-kB subunit), NF-kB2 (p52), IKK-alpha, IKK-beta, NF-kB p52/RelB, TNF-alpha \| \| 31 \| Activation of TNF-alpha-dependent pro-tumoral effect in colorectal cancer \| 9.06E-09 \| IL-6, I-kB, c-IAP2, VCAM1, RelA (p65 NF-kB subunit), GRO-1, NF-kB, G-protein alpha-s, ICAM1, NF-kB p65/c-Rel, CCL2, IKK-alpha, IL-8, IKK-beta, TNF-alpha \| \| 32 \| Glucocorticoids-mediated inhibition of pro-constrictory and pro-inflammatory signaling in airway smooth muscle cells \| 9.06E-09 \| IL-6, RelA (p65 NF-kB subunit), GRO-1, NF-kB, G-protein alpha-s, PDE4D, IRF1, NCOA2 (GRIP1/TIF2), NFKBIA, GRK2, MRLC, Histone H4, c-Jun, MKP-1, TNF-alpha \| \| 33 \| Immune response_Role of PKR in stress-induced anti-viral cell response \| 1.17E-08 \| IL-6, I-kB, IKK (cat), RelA (p65 NF-kB subunit), NF-kB, IRF1, IL-10, IKK-alpha, IFN-alpha, IL-8, NFKBIA, IKK-beta, Caspase-3, TLR2, c-Jun, TNF-alpha \| \| 34 \| PDE4 regulation of cyto/chemokine expression in inflammatory skin diseases \| 1.23E-08 \| IL-6, IKK (cat), RelA (p65 NF-kB subunit), iNOS, IL-4, CCL2, IL-10, IL23A, PDE4, IL-8, IL-22, Adenylate cyclase, NFKBIA, TNF-alpha, 14-3-3 \| \| 35 \| Development_PEDF signaling \| 1.56E-08 \| IL-6, BFL1, CCL23, c-IAP2, IKK (cat), RelA (p65 NF-kB subunit), NF-kB, c-FLIP, hASH1, AKT(PKB), Calpain 1(mu), JunB, SOD2, IKK-alpha, c-IAP1, SHARP (SPEN), tBid, NFKBIA, Caspase-3, Bid, TNF-alpha \| \| 36 \| Release of pro-inflammatory factors and proteases by alveolar macrophages in asthma \| 1.63E-08 \| GRO-2, IL-6, GRO-1, NF-kB, iNOS, IRF1, NF-kB p65/c-Rel, CCL2, MMP-1, IL-8, MMP-9, GM-CSF, TLR2, TNF-alpha \| \| 37 \| Inflammatory response in ischemia-reperfusion injury during myocardial infarction \| 2.06E-08 \| IL-6, NF-kB, HMGB1, ICAM1, CCL2, HSP70, IL-8, PSGL-1, TLR2, TNF-alpha \| \| 38 \| Immune response_IL-11 signaling pathway via MEK/ERK and PI3K/AKT cascades \| 2.18E-08 \| IL-6, I-kB, Cyclin D1, IKK (cat), RelA (p65 NF-kB subunit), IL-4, AKT(PKB), ICAM1, IL-10, SFK, IKK-alpha, IL-8, NFKBIA, Caspase-3, IL-11, c-Jun, TNF-alpha \| \| 39 \| Immune response_HMGB1/RAGE signaling pathway \| 2.98E-08 \| IL-6, I-kB, VCAM1, NF-kB, iNOS, AKT(PKB), HMGB1, ICAM1, PLAT (TPA), IL-8, NFKBIA, TLR2, c-Jun, TNF-alpha, RAGE \| \| 40 \| Immune response_CD28 signaling \| 4.59E-08 \| I-kB, AP-1, IKK (cat), RelA (p65 NF-kB subunit), Lck, NF-kB, Slp76, IL-4, AKT(PKB), c-Rel (NF-kB subunit), NF-kB p65/c-Rel, NF-kB2 (p52), IKK-alpha, IL-8, NF-kB p52/p65, GM-CSF, IKK-beta, c-Jun, CARD11 \| \| 41 \| Role of Apo-2L(TNFSF10) in Prostate Cancer cell apoptosis \| 4.68E-08 \| TRUNDD(TNFRSF10D), I-kB, c-IAP2, DR5(TNFRSF10B), c-FLIP(Long), IKK (cat), NF-kB, DR4(TNFRSF10A), c-IAP1, tBid, Caspase-3, Bid \| \| 42 \| Release of pro-inflammatory mediators and elastolytic enzymes by alveolar macrophages in COPD \| 4.69E-08 \| CHI3L1, IL-6, Matrilysin (MMP-7), RelA (p65 NF-kB subunit), CCL2, MMP-1, IL-8, MMP-9, GM-CSF, TLR2, TNF-alpha \| \| 43 \| Glucocorticoid- and LABA-mediated inhibition of pro-inflammatory signaling in airway fibroblasts/myofibroblasts \| 4.69E-08 \| IL-6, AP-1, VCAM1, NF-kB, G-protein alpha-s, IL-4, ICAM1, IL-8, GM-CSF, c-Jun, TNF-alpha \| \| 44 \| CHDI_Correlations from replication data_Causal network (positive correlations) \| 5.17E-08 \| Pyk2(FAK2), I-kB, CD83, Lck, CD40(TNFRSF5), NF-kB, Slp76, AKT(PKB), ICAM1, CD44, HSP70, IKK-alpha, SDF-1, IKK-beta, Caspase-3, TLR2, c-Jun, IP3 receptor \| \| 45 \| Signal transduction_S1P2 receptor activation signaling \| 6.55E-08 \| HB-EGF, EGR1, Cyclin D1, AP-1, VCAM1, GRO-1, NF-kB, MLC2, AKT(PKB), PLC-beta, ICAM1, CCL2, S1P2 receptor, NFKBIA, IKK-beta, MRLC, LARG, c-Jun, IP3 receptor, LIF, TNF-alpha \| \| 46 \| Interleukins-induced inflammatory response in asthmatic airway fibroblasts \| 6.8E-08 \| GRO-2, IL-6, VCAM1, RelA (p65 NF-kB subunit), GRO-1, NF-kB, ICAM1, CCL2, IL-8, GM-CSF, IL-11, c-Jun \| \| 47 \| Immune response_HMGB1/TLR signaling pathway \| 9.73E-08 \| IL-6, I-kB, E2N(UBC13), RelA (p65 NF-kB subunit), HMGB1, IKK-alpha, IFN-alpha, IL-8, IKK-beta, TLR2, TNF-alpha, RAGE \| \| 48 \| HBV-dependent NF-kB and PI3K/AKT pathways leading to HCC \| 1E-07 \| Pyk2(FAK2), I-kB, Cyclin D1, RelA (p65 NF-kB subunit), NF-kB, iNOS, AKT(PKB), NF-kB p65/c-Rel, NF-kB2 (p52), IKK-alpha, NF-kB p52/p65, MMP-9, NFKBIA, PLAU (UPA) \| \| 49 \| NF-kB-, AP-1- and MAPKs-mediated pro-inflammatory cytokine production by eosinophils in asthma \| 1.06E-07 \| IL-6, ST2(L), AP-1, GRO-1, CCL17, NF-kB, IL-4, CCL2, IL-8, IL-17F, GM-CSF, c-Jun, TNF-alpha \| \| 50 \| Signal transduction_NF-kB activation pathways \| 1.32E-07 \| I-kB, c-IAP2, NF-kB2 (p100), IKK (cat), RelA (p65 NF-kB subunit), NF-kB, RelB (NF-kB subunit), NF-kB2 (p52), IKK-alpha, c-IAP1, IKK-beta, NF-kB p52/RelB, TLR2, TNF-alpha \| |
| --- | --- | --- | --- | --- | --- | --- | --- | --- | --- | --- | --- | --- | --- | --- | --- | --- | --- | --- | --- | --- | --- | --- | --- | --- | --- | --- | --- | --- | --- | --- | --- | --- | --- | --- | --- | --- | --- | --- | --- | --- | --- | --- | --- | --- | --- | --- | --- | --- | --- | --- | --- | --- | --- | --- | --- | --- | --- | --- | --- | --- | --- | --- | --- | --- | --- | --- | --- | --- | --- | --- | --- | --- | --- | --- | --- | --- | --- | --- | --- | --- | --- | --- | --- | --- | --- | --- | --- | --- | --- | --- | --- | --- | --- | --- | --- | --- | --- | --- | --- | --- | --- | --- | --- | --- | --- | --- | --- | --- | --- | --- | --- | --- | --- | --- | --- | --- | --- | --- | --- | --- | --- | --- | --- | --- | --- | --- | --- | --- | --- | --- | --- | --- | --- | --- | --- | --- | --- | --- | --- | --- | --- | --- | --- | --- | --- | --- | --- | --- | --- | --- | --- | --- | --- | --- | --- | --- | --- | --- | --- | --- | --- | --- | --- | --- | --- | --- | --- | --- | --- | --- | --- | --- | --- | --- | --- | --- | --- | --- | --- | --- | --- | --- | --- | --- | --- | --- | --- | --- | --- | --- | --- | --- | --- | --- | --- | --- | --- | --- | --- | --- | --- | --- | --- | --- |

**Table A4. Top 50 up-regulated pathways in the MetaCore analysis of potential Endogenous Metabolic Networks. Comparison between monkeypox - infected Macaca mulatta kidney epithelial (MK2) cells and the mock controls from the GSE21001 datasets.**

| Enrichment by Metabolic Networks (Endogenous) | | | |
| --- | --- | --- | --- |
| # | Networks | pValue | Network Objects from Active Data |
| 1 | phosphatidylethanolamine pathway | 4.05E-04 | COASY, PLAP-like, CD44, PLAT (TPA), Kallikrein 1, LDLR, MMP-9, PLAU (UPA), Tissue kallikreins, LRP1, Kallikrein 3 (PSA) |
| 2 | N-acyl-sphingosine phosphate pathway | 1.89E-03 | ITGA6, Galpha(q)-specific peptide GPCRs, PP2A catalytic, CD14, PLAT (TPA), MMP-1, HNF1-alpha, Adenylate cyclase, PLAU (UPA), LRP1, eNOS |
| 3 | Lyso-Phosphatidylserine pathway | 2.46E-03 | CACNA1C, SPTBN(spectrin1-4), Neurexin 1-alpha, EPB41, Neurexin alpha, P/Q-type calcium channel alpha-1A subunit, Membrin, CASKIN1, Caspase-3 |
| 4 | 1-alkyl-glycerol_3-phosphoethanolamine pathway | 2.66E-03 | PTAFR, AKT(PKB), ICAM1, CCL2, c-Jun |
| 5 | 2-arachidonoyl-glycerol_3-phosphocholine pathway | 4.78E-03 | G-protein alpha-t, KCNK4, PLRP2, OATP-C, G-protein alpha-s, SUR1, SUR, SLC6A11, Tissue kallikreins, Caspase-3, LCAT, RAGE |
| 6 | 1-oleoyl-glycerol_3-phosphate pathway | 6.66E-03 | KCNK4, Galpha(q)-specific EDG GPCRs, AP-1, BARK, Citron, c-Jun/c-Jun, HNF1-alpha, GRK2, c-Jun, Galpha(i)-specific EDG GPCRs, GPD1 |
| 7 | 2-oleoyl-glycerol_3-phosphate pathway | 6.99E-03 | KCNK4, PLRP2, Galpha(q)-specific EDG GPCRs, CEL, LRP1, Galpha(i)-specific EDG GPCRs |
| 8 | Aminoacid metabolism_Asparagine, Aspartic acid metabolism and transport | 7.74E-03 | RelA (p65 NF-kB subunit), ADSSL1, NF-kB, iNOS, BUP1, NF-kB p65/c-Rel, ADSS, NF-kB p52/p65, NF-kB p65/p65, eNOS |
| 9 | 1-alkyl-2-lyso-glycerophosphocholine pathway | 1.04E-02 | HSP105, PTAFR, AKT(PKB), CCL2, HNF1-alpha, c-Jun |
| 10 | Phosphatidylinositol-4,5-diphosphate pathway | 1.92E-02 | Tiam1, SPTBN(spectrin1-4), ORP-family, CENTG2, PLC-beta, ICAM1, Dynamin-2, GRK2 |
| 11 | Sphingomyelin pathway | 2.56E-02 | DMBT1, PLC-beta, PHL1, NCOA2 (GRIP1/TIF2), Annexin III, LRP1, TLR2, PLC-beta2 |
| 12 | 1-oleoyl-sn-glycero-3-phosphocholine pathway | 3.33E-02 | KCNK4, PLRP2, Antithrombin III, G-protein alpha-s, SUR1, Decorin, ETS, SLC6A11, LCAT |
| 13 | [O-hexadecanoyl-(L)-carnitine pathway | 4.54E-02 | G-protein alpha-t, Transducin, rod-specific, Adenylate cyclase type VIII, G-protein alpha-s, RASA2, LARG |
| 14 | Sucrose pathway | 4.68E-02 | DMBT1, HNF1, G6PE, LRP1, Laforin, TLR2, Prolactin receptor |
| 15 | Phosphatidylinositol-3,4-diphosphate pathway | 5.06E-02 | CENTG2, AKT(PKB), Lpd |
| 16 | Ceramide pathway | 5.98E-02 | PP2A catalytic, CD14, HNF1-alpha, Adenylate cyclase, eNOS, TNF-alpha |
| 17 | Lactosylceramide pathway | 7.42E-02 | Glycolipid transfer protein, B3GN6, B3GNT5, PP2A catalytic, CD14, Adenylate cyclase, eNOS |
| 18 | (L)-leucine pathways and transport | 9.17E-02 | SLC7A8, ALPP, PP2A catalytic, IAP, AKT(PKB), PLAP-like |
| 19 | Phosphatidylinositol-3,4,5-triphosphate pathway | 9.44E-02 | ARHGEF4, Hrs, AKT(PKB), Dynamin, Dynamin-2 |
| 20 | Phosphatidylcholine pathway | 1.02E-01 | LIG-1, COASY, PHL1, HSP70, Jagged1, LRP1, LCAT, ALOX5 |
| 21 | Steroid metabolism_Cholesterol biosynthesis | 1.04E-01 | SC4MOL, HMGCS2, HMGCS1, ERG1, HMDH, DHC24 |
| 22 | 1-acyl-glycerol_3-phosphocholine pathway | 1.04E-01 | KCNK4, Pyk2(FAK2), PLRP2, OATP-C, LCAT, RAGE |
| 23 | 1-docosahexaenoyl-glycerol_3-phosphocholine pathway | 1.05E-01 | G-protein alpha-t, KCNK4, PLRP2, Antithrombin III, G-protein alpha-s, SUR1, SLC6A11, LCAT |
| 24 | Maltohexaose pathways and transport | 1.08E-01 | PAX3, G6PE, PP2A catalytic, BMP1, PLC-beta, P2Y6, Laforin, Prolactin receptor |
| 25 | 1,2-didocosapentaenoyl-sn-glycerol_3-phosphate pathway | 1.13E-01 | ORP-family, Lck, Tubulin alpha, CENTG2, PLC-beta, PHL1 |
| 26 | GalNAcbeta1-3Gal pathway | 1.13E-01 | G-protein alpha-t, Galpha(i)-specific peptide GPCRs, Galpha(q)-specific peptide GPCRs, G6PE, IL-8, LDLR |
| 27 | (L)-phenylalanine pathways and transport | 1.26E-01 | SLC7A8, GCH1, FARS2, ALPP, IAP, PLAP-like, PLC-beta |
| 28 | Maltopentaose pathways and transport | 1.27E-01 | PAX3, G6PE, PP2A catalytic, BMP1, PLC-beta, P2Y6, Laforin, Prolactin receptor |
| 29 | Alpha-L-fucosyl-(1-2)-D-galactose pathway | 1.41E-01 | COMP, Alpha 1-antitrypsin, PLAU (UPA), Tissue kallikreins, LRP1, Kallikrein 3 (PSA) |
| 30 | 1,2-didocosahexaenoyl-sn-glycerol_3-phosphate pathway | 1.46E-01 | ORP-family, Lck, Tubulin alpha, CENTG2, PLC-beta, PHL1 |
| 31 | 1-icosatrienoyl-sn-glycero-3-phosphocholine pathway | 1.66E-01 | G-protein alpha-t, KCNK4, PLRP2, G-protein alpha-s, SUR1, SUR, LCAT |
| 32 | N-acetyl-D-galactosamine pathway | 1.81E-01 | DMBT1, LRP1, TLR2 |
| 33 | L-glutamate pathways and transport | 2.07E-01 | SLC7A8, GRM2, Ionotropic glutamate receptor, NAD synthetase 1, Galpha(i)-specific metabotropic glutamate GPCRs, PPAT, Kainate receptor |
| 34 | Carbohydrate metabolism_Sucrose metabolism and transport | 2.12E-01 | HNF1, G6PE, CEACAM1, IL-4, PHL1, HNF1-alpha |
| 35 | 1-hexadecanoyl-glycerol_3-phosphate pathway | 2.16E-01 | KCNK4, Galpha(q)-specific EDG GPCRs, NRSF, Galpha(i)-specific EDG GPCRs, GPD1 |
| 36 | L-arginine pathways and transport | 2.56E-01 | PP2A catalytic, iNOS, VDR, eNOS |
| 37 | 1,2-dioleoyl-sn-glycerol_3-phosphate pathway | 2.57E-01 | ORP-family, Tubulin alpha, CENTG2, PLC-beta, PHL1 |
| 38 | 1-linoleoyl-glycerol_3-phosphate pathway | 2.64E-01 | KCNK4, Galpha(q)-specific EDG GPCRs, Citron, Galpha(i)-specific EDG GPCRs, GPD1 |
| 39 | (L)-valine pathways and transport | 2.85E-01 | SLC7A8, AP-1, HSPC124, PLC-beta, ICAM1 |
| 40 | (S)-citrulline pathway | 2.98E-01 | iNOS, Neuroglobin, Caspase-3, eNOS |
| 41 | Lipid metabolism_Triacylglycerol metabolism | 3.00E-01 | PLRP2, ALPP, CEL, GPD2, GPD1 |
| 42 | 1-palmitoyl-sn-glycero-3-phosphocholine pathway | 3.65E-01 | KCNK4, Pyk2(FAK2), SHH, LCAT |
| 43 | Glucosylceramide pathways and transport | 3.65E-01 | KCNK4, Pyk2(FAK2), SHH, LCAT |
| 44 | Glucose pathway | 3.72E-01 | ALPP, G6PE, DHSO, VDR, HNF1-alpha, Cathepsin K |
| 45 | Carbohydrate metabolism_Glycolisys, Glucogenesis and glucose transport | 3.72E-01 | ALDOB, KPYR, DHSO, GPD2, PKM2, GPD1 |
| 46 | Decanoylcarnitine pathway | 3.74E-01 | TLE, ACACB, HNF3-beta, HNF3 |
| 47 | Phosphatidic acid pathway | 3.86E-01 | PHL1 |
| 48 | Glycine pathways and transport | 4.11E-01 | SLC7A8, EGR1, GART, Pitx1, SARDH |
| 49 | Lipid metabolism_Blood group glycolipid-lactoseries metabolism | 4.11E-01 | G3ST3, B3GNT5, B3GT5 |
| 50 | Lauroylcarnitine pathway | 4.16E-01 | TLE, ACACB, HNF3-beta, HNF3 |

**Table A5. Top 50 upregulated pathways in the MetaCore analysis of potential maps. Comparison between monkeypox-infected human epithelial (HeLa) cells and the mock controls from the GSE36854 dataset.**

| # | Maps | pValue | Network Objects from Active Data |
| --- | --- | --- | --- |
| 1 | Immune response_IL-1 signaling pathway | 2.8E-21 | CCL5, COX-2 (PTGS2), IL-1 beta, IL-6, I-kB, EGR1, TPL2(MAP3K8), c-IAP2, PI3K cat class IA, FGF2, AP-1, IL-1 alpha, NF-kB2 (p100), GRO-1, MMP-13, MCPIP, NF-kB, NF-kB1 (p105), NF-kB1 (p50), ICAM1, MEKK1(MAP3K1), CCL2, NF-kB2 (p52), RANKL(TNFSF11), MMP-1, IL-8, ZFP36(Tristetraprolin), GM-CSF, PLAU (UPA), NF-kB p52/RelB, IL1RAP, CCL7, TNF-alpha |
| 2 | Inflammatory mechanisms of pancreatic cancerogenesis | 8.96E-19 | AGTR1, CCL5, COX-2 (PTGS2), IL-1 beta, C/EBP, IL-6, Bcl-XL, TNF-R2, VEGF-A, CCL20, AP-1, IL-1 alpha, GRO-1, CD40(TNFRSF5), NF-kB, c-Fos, ICAM1, CCL2, IL8RA, IL-8, Mcl-1, G-protein alpha-i family, TLR4, SDF-1, NFKBIA, PLAU (UPA), IL8RB, TNF-alpha |
| 3 | G protein-coupled receptors signaling in lung cancer | 4.84E-17 | CCL5, HB-EGF, Bcl-XL, PGE2R4, I-kB, Galpha(i)-specific peptide GPCRs, VEGF-A, Cyclin D1, Amphiregulin, BDKRB2, Galpha(q)-specific peptide GPCRs, EDNRB, G-protein alpha-s, SSTR2, c-Fos, G-protein alpha-q/11, LPAR1, GRP(1-27), IL8RA, c-Src, IL-8, HB-EGF(mature), G-protein alpha-i family, PKA-cat (cAMP-dependent), SDF-1, NTSR1, EDNRA, IL8RB |
| 4 | Immune response_Plasmin signaling | 5.1E-16 | COX-2 (PTGS2), Plasminogen, IL-1 beta, IL-6, EGR1, CCL20, IL-1 alpha, Plasmin, CD40(TNFRSF5), NF-kB, PKC, c-Fos, NF-kB1 (p50), Tissue factor, ICAM1, CCL2, PLAT (TPA), MMP-1, ITGB3, SHP-2, p38 MAPK, Histone H2B, NFKBIA, PLAU (UPA), FosB, TNF-alpha |
| 5 | Immune response_CD40 signaling in dendritic cells, monocytes, and macrophages | 1.1E-15 | Syk, COX-2 (PTGS2), IL-1 beta, IL-6, Bcl-XL, STAT5A, IDO1, TPL2(MAP3K8), c-IAP2, MHC class II, PI3K cat class IA, CD83, IL-1 alpha, NF-kB2 (p100), CD40(TNFRSF5), NF-kB, NF-kB p50/RelB, NF-kB2 (p52), IL-10, PKC-delta, IL-8, CalDAG-GEFII, p38 MAPK, IL-12 alpha, NFKBIA, NF-kB p52/RelB, TNF-alpha |
| 6 | Interleukins-induced inflammatory response in asthmatic airway fibroblasts | 1.68E-14 | CCL5, COX-2 (PTGS2), GRO-2, IL-1 beta, IL-6, IL-17, IL-1 alpha, GRO-1, NF-kB, Eotaxin, c-Fos, ICAM1, CCL2, G-CSF, IL-8, p38 MAPK, GM-CSF, IL-11 |
| 7 | TNF-alpha-induced inflammatory signaling in normal and asthmatic airway epithelium | 1.07E-13 | CCL5, IL-6, I-kB, IL-17, GRO-1, NF-kB, IL-4, Eotaxin, c-Fos, ICAM1, MEKK1(MAP3K1), CCL2, TSLP, IL-8, p38 MAPK, GM-CSF, NFKBIA, TNF-alpha |
| 8 | NF-kB-, AP-1- and MAPKs-mediated pro-inflammatory cytokine production by eosinophils in asthma | 1.07E-13 | IL-1 beta, IL-6, ST2(L), AP-1, IL-17, Myeloblastin, GRO-1, NF-kB, IL-4, Eotaxin, PRG2, CCL2, TSLP, IL-8, Leptin, PAR2, p38 MAPK, GM-CSF, TNF-alpha |
| 9 | Immune response_CD40 signaling in B cells | 1.08E-13 | Syk, IL-6, Bcl-XL, BFL1, I-kB, TPL2(MAP3K8), c-IAP2, PI3K cat class IA, AP-1, NF-kB2 (p100), CD40(TNFRSF5), NF-kB, IGH@, NF-kB1 (p50), ICAM1, NF-kB p50/RelB, MEKK1(MAP3K1), NF-kB2 (p52), c-IAP1, p38 MAPK, IGHA1, IGHG1, NFKBIA, NF-kB p52/RelB, TNF-beta, VAV-2 |
| 10 | Immune response_IL-17 signaling pathways | 1.28E-13 | COX-2 (PTGS2), IL-1 beta, IL-6, I-kB, PI3K cat class IA, CCL20, IL-17, GRO-1, NF-kB, c-Fos, ICAM1, CCL2, RANKL(TNFSF11), IL-21, G-CSF, MMP-1, IL-8, NGAL, p38 MAPK, Mucin 5AC, GM-CSF, CCL7 |
| 11 | Role of IL-8 in melanoma | 5.57E-13 | IL-1 beta, IL-6, E-cadherin, VEGF-A, NF-kB, NF-kB1 (p50), ITGB2, ICAM1, IL8RA, IL-8, p38 MAPK, ZFP36(Tristetraprolin), G-protein alpha-i family, PKA-cat (cAMP-dependent), NFKBIA, IL8RB, TNF-alpha |
| 12 | IL-1 signaling in melanoma | 9.2E-13 | COX-2 (PTGS2), GRO-2, IL-1 beta, IL-6, I-kB, VEGF-A, AP-1, IL-1 alpha, GRO-1, NF-kB, ICAM1, SOD2, CCL2, MMP-1, IL-8, GRO-3, MITF, PD-L1 |
| 13 | Macrophage and dendritic cell phenotype shift in cancer | 1.04E-12 | NF-kB p50/p50, COX-2 (PTGS2), IL-1 beta, IL-6, PGE2R4, I-kB, IDO1, MHC class II, TLR7, CD40(TNFRSF5), NF-kB, IL-4, IRE1, CSF1, IL-4R type I, PLGF, IL-10, Calcineurin B (regulatory), p38 MAPK, IL-12 alpha, TLR4, GM-CSF, SOCS3, IRF5, TLR2, SOCS1, TNF-alpha |
| 14 | B-regulatory cells and tumor cells intercellular interaction | 1.72E-12 | IL-1 beta, LTB, IL-6, ST2(L), TNF-R2, VEGF-A, OX40(TNFRSF4), TLR7, CD40(TNFRSF5), NF-kB, IL-4, IL4RA, CCL2, IL-10, IL-21, p38 MAPK, IL-12 alpha, TLR4, GM-CSF, PD-L1, IL27RA, TNF-alpha |
| 15 | Th2 cytokine- and TNF-alpha-induced inflammatory response in asthmatic airway fibroblasts | 5.09E-12 | CCL5, COX-2 (PTGS2), IL-6, CD40(TNFRSF5), NF-kB, IL-4, IL4RA, Eotaxin, c-Fos, ICAM1, CCL2, IL-4R type I, G-CSF, IL-8, GM-CSF, TNF-alpha |
| 16 | Role of fibroblasts and keratinocytes in the elicitation phase of allergic contact dermatitis | 6.4E-12 | CCL5, GRO-2, IL-1 beta, CCL20, IL-17, IL-1 alpha, GRO-1, IL-4, CCL2, IL-4R type I, IL8RA, IL-8, IL8RB, TNF-alpha |
| 17 | Immune response_Lysophosphatidic acid signaling via NF-kB | 6.52E-12 | COX-2 (PTGS2), LPAR2, IL-6, Bcl-XL, VEGF-A, CCL20, NF-kB, PKC, LPAR1, ICAM1, CCL2, TSLP, PKC-delta, c-Src, IL-8, GRO-3, A20, p38 MAPK, NFKBIA |
| 18 | TNF-alpha and IL-1 beta-mediated regulation of contraction and secretion of inflammatory factors in normal and asthmatic airway smooth muscle | 7.89E-12 | CCL5, COX-2 (PTGS2), GRO-2, IL-1 beta, IL-6, GRO-1, NF-kB, Eotaxin, Histone H3, c-Fos, PLA2, CCL2, c-Src, IL-8, GRO-3, p38 MAPK, GM-CSF, NFKBIA, Histone H4, CCL7, TNF-alpha |
| 19 | Th17 cytokines in COPD | 8.71E-12 | MMP-12, IL-1 beta, IL-6, MHC class II, CCL20, IL-17, GRO-1, NF-kB, ROR-alpha, CD8, CCL2, IL-21, IL-8, p38 MAPK, Mucin 5AC, GM-CSF |
| 20 | Cytokines/chemokines in spinal neuronal-glial interactions driving neuropathic pain | 1.21E-11 | COX-2 (PTGS2), CXCR5, IL-1 beta, IL-6, ST2(L), TNF-R2, IL-18R1, PI3K cat class IA, P2Y12, IL-17, GRO-1, NF-kB, PKC, CSF1, c-Fos, CCL2, c-Src, Cathepsin S, p38 MAPK, G-protein alpha-i family, PKA-cat (cAMP-dependent), SDF-1, C/EBPalpha, IL8RB, CXCL13, CCL7, TNF-alpha |
| 21 | Immune response_HSP60 and HSP70/ TLR signaling pathway | 1.42E-11 | IL-1 beta, IL-6, I-kB, TPL2(MAP3K8), MHC class II, IRAK1/2, CD83, AP-1, CD40(TNFRSF5), NF-kB, NF-kB1 (p105), ICAM1, IL-10, IL-8, p38 MAPK, IL-12 alpha, TLR4, TLR2, TNF-alpha |
| 22 | Neutrophil chemotaxis in asthma | 2.4E-11 | CCL5, BDKRB1, GRO-2, GRO-1, NF-kB, CCL2, PLGF, IL8RA, IL-8, GRO-3, G-protein alpha-i family, Substance P receptor, Tissue kallikreins, TLR2, IL8RB, CCL7 |
| 23 | Myeloid-derived suppressor cells and M2 macrophages in cancer | 4.96E-11 | COX-2 (PTGS2), IL-1 beta, IL-6, PGE2R4, ID1, STAT5, IDO1, VEGF-A, IL-17, NF-kB, IL-4, IL4RA, CSF1, IL-10, G-CSF, CAT-2, TLR4, GM-CSF, PD-L1, RAGE |
| 24 | IFN-gamma and Th2 cytokines-induced inflammatory signaling in normal and asthmatic airway epithelium | 6.14E-11 | CCL5, IL-2R gamma chain, IL-6, ST2(L), NF-kB, IL-4, IL4RA, Eotaxin, ICAM1, CCL2, TSLP, IL-4R type I, IL-8, p38 MAPK, SOCS3, SOCS1 |
| 25 | Renal tubulointerstitial injury in Lupus Nephritis | 6.82E-11 | CCL5, CXCR5, IL-1 beta, IL-6, I-kB, TACI(TNFRSF13B), TNF-R2, MHC class II, CD40(TNFRSF5), CSF1, ICAM1, CCL2, IL-8, HAS2, GM-CSF, PLAU (UPA), NF-kB p52/RelB, CXCL13, SOCS1, TNF-alpha |
| 26 | Maturation and migration of dendritic cells in skin sensitization | 9.58E-11 | MHC class II alpha chain, IL-1 beta, IL-6, E-cadherin, I-kB, TNF-R2, MHC class II, CD83, CD40(TNFRSF5), NF-kB, ICAM1, MHC class II beta chain, MEKK1(MAP3K1), IL-8, p38 MAPK, TNF-alpha |
| 27 | Immune response_HMGB1/RAGE signaling pathway | 9.69E-11 | IL-1 beta, IL-6, I-kB, Chromogranin A, PI3K cat class IA, IL-1 alpha, NF-kB, Tissue factor, ICAM1, PLAT (TPA), c-Src, IL-8, p38 MAPK, TLR4, NFKBIA, TLR2, TNF-alpha, RAGE |
| 28 | Immune response_Histamine H1 receptor signaling in immune response | 1.02E-10 | Histamine H1 receptor, IL-6, I-kB, MMP-13, c-Fos, G-protein alpha-q/11, Tissue factor, ICAM1, MMP-1, IL-8, Calcineurin B (regulatory), p38 MAPK, GM-CSF, NF-AT2(NFATC1), NFKBIA, eNOS, TNF-alpha |
| 29 | Expression targets of Tissue factor signaling in cancer | 1.75E-10 | PTX3, GRO-1, VEGF-C, CTGF, CSF1, Tissue factor, IL-8, Coagulation factor V, PAR2, GM-CSF, NFKBIE, PLAU (UPA) |
| 30 | PDE4 regulation of cyto/chemokine expression in arthritis | 2.2E-10 | CCL5, NF-kB p50/p50, IL-1 beta, IL-6, PGE2R4, I-kB, PI3K cat class IA, IL-17, IL-1 alpha, cAMP-GEFI, NF-kB1 (p50), CCL2, IL-10, IL-8, GM-CSF, PKA-cat (cAMP-dependent), TNF-alpha |
| 31 | Immune response_IL-4-responsive genes in type 2 immunity | 3.03E-10 | CCL5, COX-2 (PTGS2), IL-19, IL-24, CD40(TNFRSF5), IL-4, Claudin-5, IL4RA, Eotaxin, CCL13, NF-kB p50/RelB, FKHR, CCL2, IL-4R type I, IL-10, TFF3, ALOX15, Mucin 5AC, IGHG1, CCL7 |
| 32 | Release of pro-inflammatory factors and proteases by alveolar macrophages in asthma | 3.33E-10 | CCL5, GRO-2, IL-1 beta, IL-6, IL-17, GRO-1, NF-kB, IL-30, CCL2, MMP-1, IL-8, TLR4, GM-CSF, IRF5, TLR2, TNF-alpha |
| 33 | Immune response_IL-3 signaling via JAK/STAT, p38, JNK and NF-kB | 3.45E-10 | IL-6, Bcl-XL, ID1, I-kB, TACI(TNFRSF13B), STAT5A, STAT5, MHC class II, Cyclin D1, PI3K cat class IA, CD40(TNFRSF5), NF-kB, IL-4, IRE1, c-Fos, ICAM1, c-Src, c-Myc, Mcl-1, p38 MAPK, SOCS3, MKP-1, SOCS1 |
| 34 | Influence of multiple myeloma cells on bone marrow stromal cells | 3.83E-10 | COX-2 (PTGS2), IL-1 beta, IL-6, FGFR3, VEGF-A, FGF2, CD40(TNFRSF5), NF-kB, NF-kB1 (p50), ICAM1, RANKL(TNFSF11), FGFR1, IL-11, TNF-alpha |
| 35 | Immune response_IL-6 signaling pathway via JAK/STAT | 4.01E-10 | COX-2 (PTGS2), IL-6, STAT5, VEGF-A, AP-1, c-Fos, JunB, ICAM1, MEKK1(MAP3K1), FKHR, CCL2, RANKL(TNFSF11), IL-21, PKC-delta, IRS-1, Mcl-1, SHP-2, Insulin receptor, SOCS3, SOCS1 |
| 36 | Signal transduction_Adenosine A2B receptor signaling pathway | 4.01E-10 | IL-6, VEGF-A, PI3K cat class IA, G-protein alpha-s, CALDAG-GEFI, IL-4, PKC, cAMP-GEFI, NF-kB1 (p105), G-protein alpha-q/11, JunB, IL-10, cAMP-GEFII, Calcineurin B (regulatory), p38 MAPK, IL-12 alpha, NF-AT2(NFATC1), PKA-cat (cAMP-dependent), eNOS, TNF-alpha |
| 37 | Release of pro-inflammatory mediators and elastolytic enzymes by alveolar macrophages in COPD | 4.12E-10 | MMP-12, IL-1 beta, IL-6, Matrilysin (MMP-7), CCL2, MMP-1, Cathepsin S, IL-8, p38 MAPK, TLR4, GM-CSF, TLR2, TNF-alpha |
| 38 | Glucocorticoid- and LABA-mediated inhibition of pro-inflammatory signaling in airway fibroblasts/myofibroblasts | 4.12E-10 | IL-1 beta, IL-6, AP-1, IL-1 alpha, NF-kB, G-protein alpha-s, IL-4, ICAM1, IL-4R type I, IL-8, GM-CSF, PKA-cat (cAMP-dependent), TNF-alpha |
| 39 | Immune response_Th17, Th22 and Th9 cell differentiation | 4.75E-10 | IL-1 beta, IL-6, MHC class II, AP-1, IL-17, CD40(TNFRSF5), NF-kB, IL-4, ROR-alpha, IL-4R type I, IL-10, IL-21, TGF-beta receptor type II, NF-AT2(NFATC1), TNF-alpha |
| 40 | Pro-inflammatory cytokine release from eosinophils in asthma | 6.2E-10 | CCL5, IL-16 mature, IL-1 beta, IL-6, TrkA, IL-17, IL-16, IL-4, Eotaxin, IL-8, GM-CSF, LIF, TNF-alpha, Fc alpha receptor |
| 41 | NF-kB pathway in multiple myeloma | 9.84E-10 | IL-6, Bcl-XL, I-kB, TACI(TNFRSF13B), c-IAP2, Cyclin D1, NF-kB2 (p100), CD40(TNFRSF5), NF-kB, NF-kB1 (p50), NF-kB2 (p52), c-IAP1, c-Myc, NF-kB p52/RelB |
| 42 | Immune response_T cell subsets: secreted signals | 1.22E-09 | CCL5, IL-6, CCL20, IL-17, Amphiregulin, IL-1 alpha, IL-4, IL-10, IL-21, GM-CSF, TNF-beta, TNF-alpha |
| 43 | Cigarette smoke-induced inflammatory signaling in airway epithelial cells | 1.53E-09 | COX-2 (PTGS2), HB-EGF, IL-1 beta, IL-6, Amphiregulin, ICAM1, c-Src, IL-8, GM-CSF, PKA-cat (cAMP-dependent), NFKBIA, TNF-alpha, ErbB3, sICAM1 |
| 44 | Apoptosis and survival_Anti-apoptotic TNFs/NF-kB/Bcl-2 pathway | 1.61E-09 | Bcl-XL, BFL1, I-kB, TACI(TNFRSF13B), TNF-R2, OX40(TNFRSF4), IRAK1/2, NF-kB2 (p100), CD40(TNFRSF5), NF-kB, RelB (NF-kB subunit), NF-kB2 (p52), RANKL(TNFSF11), NF-kB p52/RelB, TNF-alpha |
| 45 | Glomerular injury in Lupus Nephritis | 1.67E-09 | CCL5, GRO-2, IL-1 beta, IL-6, Cyclin D1, IL-17, GRO-1, NF-kB, CSF1, ICAM1, CCL2, C3a, MMP-1, IL-8, HAS2, NGAL, GRO-3, A20, p38 MAPK, GM-CSF, LIN-28, TNF-alpha |
| 46 | Immune response_IL-10 signaling pathway | 1.77E-09 | COX-2 (PTGS2), IL-1 beta, IL-6, Bcl-XL, IKBZ, STAT5, MHC class II, PI3K cat class IA, IL-1 alpha, NF-kB, NF-kB1 (p50), ICAM1, IL-10, G-CSF, IL-8, GM-CSF, SOCS3, TNF-alpha |
| 47 | Inflammatory response in ischemia-reperfusion injury during myocardial infarction | 1.84E-09 | IL-1 beta, IL-6, NF-kB, ICAM1, CCL2, IL8RA, C3a, IL-8, TLR4, TLR2, TNF-alpha |
| 48 | Immune response_Generation of memory CD4+ T cells | 2.35E-09 | Bcl-XL, BFL1, STAT5, MHC class II, OX40(TNFRSF4), AP-1, IL-7, NF-kB, IL-4, IL4RA, FKHR, IL-4R type I, Mcl-1, NF-AT2(NFATC1) |
| 49 | Fibroblast/ myofibroblast proliferation in asthmatic airways | 2.35E-09 | BDKRB1, Histamine H1 receptor, IL-1 beta, PI3K cat class IA, EDNRB, NF-kB, CTGF, PKC, Tissue factor, ITGB3, TGF-beta receptor type II, PAR2, EDNRA, FGFR1 |
| 50 | Immune response_IL-18 signaling | 2.36E-09 | COX-2 (PTGS2), IL-1 beta, IL-6, I-kB, IL-18R1, PI3K cat class IA, AP-1, IL-1 alpha, NF-kB, c-Fos, ICAM1, CCL2, c-Src, IL-8, p38 MAPK, IL18RAP, Bcl-XS, TNF-alpha |

**Table A6. Top 50 upregulated pathways in the MetaCore analysis of potential Endogenous Metabolic Networks. Comparison between monkeypox-infected human epithelial (HeLa) cells and the mock controls from the GSE36854 datasets.**

| # | Networks | pValue | Network Objects from Active Data |
| --- | --- | --- | --- |
| 1 | phosphatidylethanolamine pathway | 4.05E-04 | COASY, PLAP-like, CD44, PLAT (TPA), Kallikrein 1, LDLR, MMP-9, PLAU (UPA), Tissue kallikreins, LRP1, Kallikrein 3 (PSA) |
| 2 | N-acyl-sphingosine phosphate pathway | 1.89E-03 | ITGA6, Galpha(q)-specific peptide GPCRs, PP2A catalytic, CD14, PLAT (TPA), MMP-1, HNF1-alpha, Adenylate cyclase, PLAU (UPA), LRP1, eNOS |
| 3 | Lyso-Phosphatidylserine pathway | 2.46E-03 | CACNA1C, SPTBN(spectrin1-4), Neurexin 1-alpha, EPB41, Neurexin alpha, P/Q-type calcium channel alpha-1A subunit, Membrin, CASKIN1, Caspase-3 |
| 4 | 1-alkyl-glycerol_3-phosphoethanolamine pathway | 2.66E-03 | PTAFR, AKT(PKB), ICAM1, CCL2, c-Jun |
| 5 | 2-arachidonoyl-glycerol_3-phosphocholine pathway | 4.78E-03 | G-protein alpha-t, KCNK4, PLRP2, OATP-C, G-protein alpha-s, SUR1, SUR, SLC6A11, Tissue kallikreins, Caspase-3, LCAT, RAGE |
| 6 | 1-oleoyl-glycerol_3-phosphate pathway | 6.66E-03 | KCNK4, Galpha(q)-specific EDG GPCRs, AP-1, BARK, Citron, c-Jun/c-Jun, HNF1-alpha, GRK2, c-Jun, Galpha(i)-specific EDG GPCRs, GPD1 |
| 7 | 2-oleoyl-glycerol_3-phosphate pathway | 6.99E-03 | KCNK4, PLRP2, Galpha(q)-specific EDG GPCRs, CEL, LRP1, Galpha(i)-specific EDG GPCRs |
| 8 | Aminoacid metabolism_Asparagine, Aspartic acid metabolism and transport | 7.74E-03 | RelA (p65 NF-kB subunit), ADSSL1, NF-kB, iNOS, BUP1, NF-kB p65/c-Rel, ADSS, NF-kB p52/p65, NF-kB p65/p65, eNOS |
| 9 | 1-alkyl-2-lyso-glycerophosphocholine pathway | 1.04E-02 | HSP105, PTAFR, AKT(PKB), CCL2, HNF1-alpha, c-Jun |
| 10 | Phosphatidylinositol-4,5-diphosphate pathway | 1.92E-02 | Tiam1, SPTBN(spectrin1-4), ORP-family, CENTG2, PLC-beta, ICAM1, Dynamin-2, GRK2 |
| 11 | Sphingomyelin pathway | 2.56E-02 | DMBT1, PLC-beta, PHL1, NCOA2 (GRIP1/TIF2), Annexin III, LRP1, TLR2, PLC-beta2 |
| 12 | 1-oleoyl-sn-glycero-3-phosphocholine pathway | 3.33E-02 | KCNK4, PLRP2, Antithrombin III, G-protein alpha-s, SUR1, Decorin, ETS, SLC6A11, LCAT |
| 13 | [O-hexadecanoyl-(L)-carnitine pathway | 4.54E-02 | G-protein alpha-t, Transducin, rod-specific, Adenylate cyclase type VIII, G-protein alpha-s, RASA2, LARG |
| 14 | Sucrose pathway | 4.68E-02 | DMBT1, HNF1, G6PE, LRP1, Laforin, TLR2, Prolactin receptor |
| 15 | Phosphatidylinositol-3,4-diphosphate pathway | 5.06E-02 | CENTG2, AKT(PKB), Lpd |
| 16 | Ceramide pathway | 5.98E-02 | PP2A catalytic, CD14, HNF1-alpha, Adenylate cyclase, eNOS, TNF-alpha |
| 17 | Lactosylceramide pathway | 7.42E-02 | Glycolipid transfer protein, B3GN6, B3GNT5, PP2A catalytic, CD14, Adenylate cyclase, eNOS |
| 18 | (L)-leucine pathways and transport | 9.17E-02 | SLC7A8, ALPP, PP2A catalytic, IAP, AKT(PKB), PLAP-like |
| 19 | Phosphatidylinositol-3,4,5-triphosphate pathway | 9.44E-02 | ARHGEF4, Hrs, AKT(PKB), Dynamin, Dynamin-2 |
| 20 | Phosphatidylcholine pathway | 1.02E-01 | LIG-1, COASY, PHL1, HSP70, Jagged1, LRP1, LCAT, ALOX5 |
| 21 | Steroid metabolism_Cholesterol biosynthesis | 1.04E-01 | SC4MOL, HMGCS2, HMGCS1, ERG1, HMDH, DHC24 |
| 22 | 1-acyl-glycerol_3-phosphocholine pathway | 1.04E-01 | KCNK4, Pyk2(FAK2), PLRP2, OATP-C, LCAT, RAGE |
| 23 | 1-docosahexaenoyl-glycerol_3-phosphocholine pathway | 1.05E-01 | G-protein alpha-t, KCNK4, PLRP2, Antithrombin III, G-protein alpha-s, SUR1, SLC6A11, LCAT |
| 24 | Maltohexaose pathways and transport | 1.08E-01 | PAX3, G6PE, PP2A catalytic, BMP1, PLC-beta, P2Y6, Laforin, Prolactin receptor |
| 25 | 1,2-didocosapentaenoyl-sn-glycerol_3-phosphate pathway | 1.13E-01 | ORP-family, Lck, Tubulin alpha, CENTG2, PLC-beta, PHL1 |
| 26 | GalNAcbeta1-3Gal pathway | 1.13E-01 | G-protein alpha-t, Galpha(i)-specific peptide GPCRs, Galpha(q)-specific peptide GPCRs, G6PE, IL-8, LDLR |
| 27 | (L)-phenylalanine pathways and transport | 1.26E-01 | SLC7A8, GCH1, FARS2, ALPP, IAP, PLAP-like, PLC-beta |
| 28 | Maltopentaose pathways and transport | 1.27E-01 | PAX3, G6PE, PP2A catalytic, BMP1, PLC-beta, P2Y6, Laforin, Prolactin receptor |
| 29 | Alpha-L-fucosyl-(1-2)-D-galactose pathway | 1.41E-01 | COMP, Alpha 1-antitrypsin, PLAU (UPA), Tissue kallikreins, LRP1, Kallikrein 3 (PSA) |
| 30 | 1,2-didocosahexaenoyl-sn-glycerol_3-phosphate pathway | 1.46E-01 | ORP-family, Lck, Tubulin alpha, CENTG2, PLC-beta, PHL1 |
| 31 | 1-icosatrienoyl-sn-glycero-3-phosphocholine pathway | 1.66E-01 | G-protein alpha-t, KCNK4, PLRP2, G-protein alpha-s, SUR1, SUR, LCAT |
| 32 | N-acetyl-D-galactosamine pathway | 1.81E-01 | DMBT1, LRP1, TLR2 |
| 33 | L-glutamate pathways and transport | 2.07E-01 | SLC7A8, GRM2, Ionotropic glutamate receptor, NAD synthetase 1, Galpha(i)-specific metabotropic glutamate GPCRs, PPAT, Kainate receptor |
| 34 | Carbohydrate metabolism_Sucrose metabolism and transport | 2.12E-01 | HNF1, G6PE, CEACAM1, IL-4, PHL1, HNF1-alpha |
| 35 | 1-hexadecanoyl-glycerol_3-phosphate pathway | 2.16E-01 | KCNK4, Galpha(q)-specific EDG GPCRs, NRSF, Galpha(i)-specific EDG GPCRs, GPD1 |
| 36 | L-arginine pathways and transport | 2.56E-01 | PP2A catalytic, iNOS, VDR, eNOS |
| 37 | 1,2-dioleoyl-sn-glycerol_3-phosphate pathway | 2.57E-01 | ORP-family, Tubulin alpha, CENTG2, PLC-beta, PHL1 |
| 38 | 1-linoleoyl-glycerol_3-phosphate pathway | 2.64E-01 | KCNK4, Galpha(q)-specific EDG GPCRs, Citron, Galpha(i)-specific EDG GPCRs, GPD1 |
| 39 | (L)-valine pathways and transport | 2.85E-01 | SLC7A8, AP-1, HSPC124, PLC-beta, ICAM1 |
| 40 | (S)-citrulline pathway | 2.98E-01 | iNOS, Neuroglobin, Caspase-3, eNOS |
| 41 | Lipid metabolism_Triacylglycerol metabolism | 3.00E-01 | PLRP2, ALPP, CEL, GPD2, GPD1 |
| 42 | 1-palmitoyl-sn-glycero-3-phosphocholine pathway | 3.65E-01 | KCNK4, Pyk2(FAK2), SHH, LCAT |
| 43 | Glucosylceramide pathways and transport | 3.65E-01 | KCNK4, Pyk2(FAK2), SHH, LCAT |
| 44 | Glucose pathway | 3.72E-01 | ALPP, G6PE, DHSO, VDR, HNF1-alpha, Cathepsin K |
| 45 | Carbohydrate metabolism_Glycolisys, Glucogenesis and glucose transport | 3.72E-01 | ALDOB, KPYR, DHSO, GPD2, PKM2, GPD1 |
| 46 | Decanoylcarnitine pathway | 3.74E-01 | TLE, ACACB, HNF3-beta, HNF3 |
| 47 | Phosphatidic acid pathway | 3.86E-01 | PHL1 |
| 48 | Glycine pathways and transport | 4.11E-01 | SLC7A8, EGR1, GART, Pitx1, SARDH |
| 49 | Lipid metabolism_Blood group glycolipid-lactoseries metabolism | 4.11E-01 | G3ST3, B3GNT5, B3GT5 |
| 50 | Lauroylcarnitine pathway | 4.16E-01 | TLE, ACACB, HNF3-beta, HNF3 |
